# Children’s Headache Through Drawings: A Narrative Review and a Portrait Gallery

**DOI:** 10.3390/life15070996

**Published:** 2025-06-23

**Authors:** Floriana Ferro, Caterina Gaspari, Giulia Manfrè, Federica Cernigliaro, Daniela D’Agnano, Ruben Panzica, Edvige Correnti, Maria Rosita Ruta, Francesca Marchese, Renata Pitino, Mariarita Capizzi, Giuseppe Santangelo, Antonella Versace, Vittorio Sciruicchio, Vincenzo Raieli

**Affiliations:** 1 Child Neuropsychiatry Unit Department, Pro.MI.S.E. “G. D’Alessandro”, University of Palermo, 90127 Palermo, Italy; flofloferro@gmail.com (F.F.); caterinagaspari.pe@gmail.com (C.G.); giuliamanfre95@gmail.com (G.M.); fede.cernigliaro@gmail.com (F.C.); 2 Child Neuropsychiatry Department, Istituto Mediterraneo di Eccellenza Pediatrica, ARNAS Civico, 90127 Palermo, Italy; edvige.correnti@arnascivico.it (E.C.); renata.pitino@arnascivico.it (R.P.); mariaritacapizzi93@gmail.com (M.C.); giuseppe.santangelo@arnascivico.it (G.S.); 3 Children Epilepsy and EEG Center, San Paolo Hospital, ASL Bari, 70132 Bari, Italy; daniela.dagnano@gmail.com (D.D.); vittorio.sciruicchio@asl.bari.it (V.S.); 4 Rheumatolgy Unit, Department of Clinical and Experimental Medicine, University of Messina, 98122 Messina, Italy; rubenslashpanzica@gmail.com; 5 Child Neuropsychiary Unit-ASP 6, 90100 Palermo, Italy; rositaruta1@gmail.com (M.R.R.); marchesefrancescaa@gmail.com (F.M.); 6 Pediatric Headache Center, Pediatric Emergency Department, Regina Margherita Children’s Hospital, 10126 Turin, Italy; antoversace93@gmail.com

**Keywords:** children, headache, migraine, drawings, diagnosis, pain, nonverbal communication

## Abstract

Headache represents one of the most prevalent and disabling conditions in the pediatric population, with significant repercussions on mental and psychological well-being, as well as on academic achievement and social functioning, ultimately leading to a marked reduction in quality of life. Currently, the diagnosis of headache is based on the clinical criteria of the third edition of the International Classification of Headache Disorders (ICHD-3). However, the characteristics of headache may differ between adults and children, as well as the ability of children to provide a complete description of the pain and associated symptoms. The immature narrative skills of children can represent a limitation in defining the clinical phenotype of headache, making the diagnosis more complex. This is even more challenging when extracting information about the characteristics of the headache in children whose verbal expression is poorly developed or completely absent. Given these limitations, clinical psychology has long used drawing as an effective diagnostic instrument to bypass verbal communication barriers. This tool provides unique access to children’s psychological and emotional states, as a direct window into their inner world and as an expressive medium that often generates more detailed, accurate, and clinically actionable information, compared to verbal reports alone. For these reasons, drawing has been recognized as a valuable diagnostic tool for decades, with multiple studies demonstrating specificity and accuracy rates comparable to standard clinical assessments. Particularly for young children, drawings may give access to fundamental information that might otherwise remain inaccessible, thereby allowing both accurate diagnosis and individualized treatment planning. Multiple studies have highlighted and confirmed the graphic differences between representations of various types of headaches and the undeniable utility of an “artistic diagnosis” alongside the clinical one. Furthermore, the literature suggests and encourages the use of drawing in clinical practice, both in the diagnostic process and during subsequent follow-up, as an effective, enjoyable, easy-to-use, and low-cost resource. Accordingly, we propose a narrative review accompanied by a curated collection of drawings that may help identify and categorize specific correlations between graphic representations and clinical phenotypes, such as pain location, quality, intensity, association with nausea and vomiting, photophobia and phonophobia, and types of migraine aura. Our goal is to create a visual reference that can aid clinicians in the accurate interpretation of children’s drawings. Additionally, we aim to promote the integration of this method into routine clinical practice to improve diagnostic precision and support a more child-centered model of care. We also hope to propose new iconographic models to further enrich the diagnostic framework.

## 1. Introduction

Headache is one of the most common neurological disorders in the pediatric population, with an estimated prevalence of 88% [1]. A recent meta-analysis reported that the prevalence of primary headache is 15% for migraine and 17% for tension-type headache [2].

However, there is a lack of extensive epidemiological research on the prevalence and incidence of primary headaches in the pediatric and adolescent populations, and many studies often exhibit statistical heterogeneity. This variability arises from differences in study populations, including factors such as age range, sex, and socio-economic status, as well as from the different methodologies employed (e.g., school-based surveys, clinician interviews, telephone interviews) and the inconsistent application of diagnostic criteria, which are sometimes not specifically tailored to developmental age groups [3].

In terms of disability, headache has a strong impact on daily functioning and quality of life, affecting children’s development and functioning in school, home, or social environment, resulting in a severely debilitating condition [4].

Despite the widespread impact, migraine remains underdiagnosed and undertreated in children [2].

Currently, clinical diagnosis is based on the criteria outlined in the third edition of the International Classification of Headache Disorders (ICHD-3), which involves a comprehensive evaluation of the patient’s medical history, family and social history, physical examination, and complete neurological examination. In cases where suspicious findings or red flags are present, additional investigations such as blood tests and neuroimaging may be conducted to differentiate primary headaches (e.g., migraine and tension-type headache) from secondary headaches (e.g., tumors, head trauma, infections, idiopathic intracranial hypertension, etc.).

Moreover, the ICHD-3 criteria primarily focus on adult headache phenotypes and do not fully account for potential differences in pediatric presentations, particularly in pre-pubertal children; therefore, this limitation may contribute to delays in diagnosis and treatment. It has been suggested that pediatric migraines, especially in younger children, tend to have shorter attack durations [5,6]. The attacks are less lateralized compared to those in adults [7,8,9].

Moreover, children’s migraines tend to be slightly associated with visual aura, while constitutional symptoms such as nausea, vomiting, flushing, and pallor are more common.

Under the age of 18, migraine attacks typically last between 2 and 72 h, with clinical evidence supporting untreated attacks of shorter duration [3,5,6]. Unilateral pain generally appears in late adolescence or early adulthood [3]. Migraine headaches are usually frontotemporal, while occipital localization in children is rare and warrants careful diagnostic evaluation [3]. Additionally, in young children, photophobia and phonophobia are often inferred from behavioral observations rather than explicitly reported symptoms [10].

The mentioned clinical differences between adults and children could be the result of differences in the degree of brain maturation, including myelination, new synapse formation, and synaptic reorganization [8].

Given the high prevalence of headaches in children and the lack of age-specific diagnostic criteria, there is a clear need for accurate clinical diagnostic guidelines [2].

To increase the sensitivity of the ICHD criteria concerning childhood headaches, considering that the child is not a “small adult”, several revisions to the original criteria have been proposed. These include reducing the minimum duration of an episode from 2 h to 1 h, decreasing the number of required episodes from 5 to 3, and removing the requirement for unilaterality [3,11].

Since attack duration and laterality are key diagnostic criteria for migraines, understanding these variations in children could provide insight into whether distinct diagnostic criteria are necessary for the pediatric population [12].

Although there are no specific diagnostic tests for migraine or tension-type headache, an accurate diagnosis is essential to provide the best treatment approaches, which may differ from one type of headache to another.

The diagnosis is further complicated by children’s limited narrative abilities, especially in those with underdeveloped or absent verbal expression. To tackle the challenge of defining the clinical phenotype of headaches, clinicians often rely on parent-reported medical histories. However, their responses may not always accurately represent the child’s condition and can sometimes exaggerate or deny the symptoms [13].

Developing new strategies to overcome communication barriers is an ongoing challenge. In this context, drawings can serve as a valuable tool, especially since children are naturally expressive artists. For many, visual expression may prove more effective than verbal communication [2].

The literature [11,14,15,16,17,18,19,20] supports and encourages the use of drawings in clinical practice, both as part of the diagnostic process and during follow-up, highlighting their value as an effective, enjoyable, easy-to-use, and low-cost resource. However, relatively few studies have directly assessed the clinical utility of drawings in the diagnosis of pediatric headache, and existing research on the approach to the topic from heterogeneous perspectives in terms of methodology and objectives [11,14,15,16,17,18,19,20].

In this context, we propose a narrative review accompanied by a collection of drawings, aimed at identifying and cataloguing specific correlations between graphic representations and clinical phenotypes. These include pain location, quality, intensity, and associations with symptoms such as nausea, vomiting, photophobia, phonophobia, and types of migraine aura.

The primary goal is to establish a reference collection that helps clinicians in accurately interpreting children’s drawings, enhancing diagnostic precision and patient care. In addition, headache drawings can be used to longitudinally track clinical progression and treatment response [15].

## 2. Review of Literature

Drawing is a valuable tool that provides access to a child’s psychological and emotional world, offering a direct window into their inner thoughts and feelings. It serves both as a genuine form of expression and an effective means of communication, often exceeding verbal methods in detail, clarity, and impact.

A literature search was conducted using PUBMED, Google Scholar, and Researchgate, covering the period from 1980 to 2024. The keywords used included children, hemicrania, tension headache, headaches, pain, and drawings. We selected all the studies that analyzed, as their main purpose, the relationship between drawings and headaches in children and adolescents. Due to the limited number of studies available, we opted for a narrative synthesis to systematically discuss the literature. The selected studies are presented in chronological order.

### 2.1. Headache in Children’s Drawings

Unruh et al. [14] were among the first to explore the utility of children’s drawings as a diagnostic tool for evaluating pain, particularly in pediatric headaches. This method, based on the observation of visual and symbolic representations, provided an alternative for assessing pain where verbal communication was limited.

The authors analyzed a sample of 109 children aged 5 to 18 years, all presenting with chronic or recurrent headaches. Each child was instructed to draw their pain and themselves experiencing pain, and the resulting illustrations were systematically analyzed for specific features, including the use of colors, line characteristics, and symbolic elements. Statistical analysis revealed that 72% of the children used dark or aggressive imagery (e.g., storms, explosions, or jagged lines) to depict severe or pulsating headaches, correlating with clinical diagnoses of migraine (*p* < 0.01).

Additionally, 65% of the drawings included localized indications of pain, such as shading or emphasizing the head region, aligning with self-reported pain locations (*p* < 0.05). Emotional content was also significant: 58% of children’s drawings displayed contextual elements such as distorted figures or somber backgrounds, which were associated with higher scores on anxiety scales (*p* < 0.05). The most frequently used colors were red and black, with no significant differences found based on gender or age.

More specifically, children’s illustrations of pain could be classified into seven categories such as: actions and tools (32%), personification of pain (19%), physiological representation of pain (5%), perceptual disturbances (6%), abstract representation of pain (25%), localization (3%) and non-specific drawings (3%). Children’s illustrations of themselves experiencing pain included: the recipient of pain (11%), an agent that relieves pain (44%), emotions resulting from pain (40%), localization (3%), and non-specific drawings (only 2%).

The non-invasive and basic nature of the method facilitated its application, requiring minimal preparation and yielding high compliance rates (95%).

In longitudinal follow-up, subsequent drawings showed changes in pain representation that correlated with reported clinical improvements, highlighting the method’s value in assessing treatment efficacy.

These findings indicate that children’s drawings serve not only as a reliable diagnostic complement, but also as a valuable tool for assessing both physical and emotional dimensions of pediatric headache. When integrated into clinical practice, this technique may improve diagnostic accuracy, particularly in younger populations, while providing a more holistic understanding of pain experiences [15].

Stafstrom et al. [11] also explored the diagnostic utility of children’s drawings, highlighting significant correlations between graphic representations and clinical phenotypes. A total of 226 drawings were analyzed, recognizing the multitude of elements, information, and details emerging from the images.

The study involved children aged 4 to 18 years, recruited from specialized pediatric neurology clinics. All participants met established diagnostic criteria for primary headache disorders, such as migraines and tension-type headaches, according to the International Classification of Headache Disorders (ICHD) [10].

Each child was guided to illustrate their headache experience, depicting pain location, intensity, and associated emotional or sensory features. The resulting drawings were subsequently evaluated by a multidisciplinary team of pediatric neurologists and psychologists. For instance, 90% of children diagnosed with migraines included precise indications of pain localization in their drawings, often using shading or intense markings in the head region (*p* < 0.01). Additionally, 78% of the drawings depicted emotional elements such as dark colors or negative contexts, closely associated with concurrent anxiety symptoms (*p* < 0.05). Metaphorical elements like lightning bolts or explosions, observed in 68% of drawings, were linked to pulsating pain characteristic of migraines. In contrast, tension-type headache sufferers drew elements such as bandages and head-binding material with fewer expressions of distress. The drawings were compared with the clinical diagnosis, showing a sensitivity of 93.1%, a specificity of 82.7%, and a positive predictive value of 87.1% for migraine, thus demonstrating significant associations between the graphic details and the children’s reported experiences.

The methodology’s primary strength lay in its non-invasive nature, requiring minimal preparation while achieving excellent participant compliance (95%). In longitudinal follow-up [15], subsequent drawings provided valuable insights into the progression of the children’s conditions. For example, as treatment effectiveness increased, the drawings revealed decreased emotional intensity and simplified pain representations. This dynamic evolution reinforced the utility of drawings in monitoring treatment efficacy over time. Moreover, pediatric neurologists collaborated with psychologists to interpret symbolic and emotional content, ensuring a comprehensive understanding of the child’s experience.

This study highlighted the importance of creative approaches in pediatric neurology, offering a new dimension to symptom evaluation and patient care [11].

Other studies have further reinforced and validated the utility of distinctive drawings for various types of headaches.

Wojaczynska-Stanek et al. [16] explored the diagnostic potential of children’s drawings. The research involved 68 girls aged 5–18 and 56 boys aged 7–18. Of the 124 children, 40 had been clinically diagnosed with migraine, 47 with tension-type headache, and 37 with “other” types. The authors analyzed the children’s pain drawings to uncover patterns that could enhance diagnostic precision and deepen insight into the subjective experience of pediatric headaches.

The findings revealed that 68% of the drawings included specific pain localization, with notable differences between migraine and tension-type headache. Children with migraine commonly depicted pain as localized to one side of the head or around the eyes (78%), while those with tension-type headache illustrated a diffuse or generalized pressure-like sensation across the entire head (64%). Additionally, 25% of the drawings featured symbolic elements, such as tears, lightning bolts, or storm clouds, highlighting the emotional distress linked to their headaches. In contrast, 7% lacked specific pain-related depictions and instead showed a general sense of discomfort.

Color choice also emerged as a significant aspect of the drawings, reflecting the children’s emotional responses to their condition. Black, gray, and dark blue predominated in 73% of the drawings, often symbolizing intense or negative feelings, while 27% incorporated brighter hues, suggesting a less distressing or neutral perception of the pain.

The study confirms the utility of children’s drawings as a complementary tool in the clinical assessment of headaches [16].

Mosquera and Martino [17] analyzed children’s and adolescents’ drawings to identify migraine in headache patients. The study involved 48 patients aged 5 to 19 years diagnosed with headache. Each child was asked to draw their perception of the pain, focusing on its location, intensity, and associated symptoms. The drawings were independently evaluated by two pediatric neurologists: one performing an artistic assessment, the other a clinical diagnosis. This protocol assessed the predictive accuracy of artistic evaluation relative to clinical findings. The study revealed that 75% of the drawings depicted pain as unilateral, primarily in the orbital or temporal regions, consistent with the typical characteristics of migraines. In 20% of cases, the drawings showed generalized or bilateral pain, often associated with less severe or atypical migraines, while 5% of the drawings lacked specific pain localization but included abstract or emotional elements to express discomfort. Symbolic elements, such as lightning bolts, dark clouds, or sharp lines, were present in 60% of the drawings, reflecting the intensity of the pain. Emotional imagery, such as tears or exaggerated expressions, appeared in 30% of the cases, whereas 10% of the drawings were minimalistic but still conveyed the perception of pain. The color choice was another significant aspect, with colors like black, gray, and dark blue dominating 80% of the drawings, correlating with severe or distressing headache experiences. Bright colors appeared in 20% of the drawings, often associated with milder or less disabling headaches. The artistic diagnosis accurately predicted the clinical diagnosis of migraine with a sensitivity of 69.6%, specificity of 88%, positive predictive value of 84.2%, and a negative predictive value of 75.9%, demonstrating a strong concordance between the visual analysis of the drawings and the clinical evaluation based on IHS criteria. However, in 15% of the cases, the artistic diagnosis either underestimated or overestimated the severity of the migraine or failed to identify atypical features [18].

A larger study conducted by Mazzotta et al. [18] involved 67 children aged 6 to 14 years who experienced recurrent headaches, along with a control group of 90 healthy children. The drawing of headaches was analyzed by two child neuropsychiatrists blinded to clinical data, in order to differentiate migraine from tension-type headache.

Of the headache drawings, 78% included specific pain localization. Among these, 56% depicted unilateral or focal pain typically associated with migraine, while 22% illustrated bilateral or diffuse pain, more commonly linked to tension-type headaches. In the remaining 22% of the drawings, precise pain localization was absent, but these still provided valuable insights through symbolic or emotional representations. Almost half of the drawings (48%) contained symbolic imagery, such as lightning bolts, dark clouds, or tears, highlighting the intensity and emotional burden of the headaches. Additionally, 32% included visual cues like zigzag patterns or wavy lines, often indicative of migraine aura or light sensitivity. The use of colors, such as black, gray, or dark blue, was prevalent in 84% of the drawings, correlating with severe or distressing headache experiences, while brighter or neutral colors appeared in 16% of cases, typically reflecting milder symptoms.

The study found that 55% of the participants were clinically diagnosed with migraine, while 45% were classified with tension-type headache. For migraine, the sensitivity, specificity, and positive predictive value (PPV) of the drawings were 85.71%, 81.48%, and 85.71%, respectively. For tension-type headache, the sensitivity, specificity, and PPV were 81.48%, 85.71%, and 81.48%, respectively.

The study demonstrated a strong correspondence between artistic and clinical diagnoses, with an accuracy of 83%. Among children with migraines, the artistic diagnosis was correct in 88% of cases, while for those with tension-type headaches, accuracy was 76%. However, 17% of the cases showed discrepancies, often due to atypical or overlapping headache features [18].

Yilmaz et al. [19] explored how drawings could enhance the diagnostic process in adolescents aged 14 to 18 diagnosed with migraine with visual aura. Five participants (three girls and two boys) were included. Visual aura was observed in all participants. The depicted visual disturbances included blurry vision, bright lights, zigzag lines, and scotomas. These representations helped overcome communication barriers often encountered in pediatric neurology, providing clinicians with a more comprehensive understanding of the patients’ experiences. The study emphasized that drawing symptoms can support both diagnostic and follow-up processes, as visual descriptions are sometimes challenging for younger patients to articulate.

Another transversal and descriptive study conducted by Garcia-Ron et al. [20] assessed the concordance between the “artistic” diagnosis of headache and the clinical diagnosis.

A neuropediatrician and two neurologists, experts in headache, were asked to review the patient’s drawing in order to formulate the artistic diagnosis, while the clinical diagnosis was made after a complete anamnesis and a clinical examination by a different neuropediatrician.

The study included 132 patients (61.1%, girls; mean age, 12 years) with clinical diagnoses of migraine (59.1%), tension-type headache (38.2%), and other headaches (trigeminal-autonomic and nummular) accounting for 2.7%. Each patient was asked to make a single drawing illustrating their own headache, without any suggestion or additional instruction, and without any time limit.

The drawings most frequently depicted symptoms related to pain localization, often highlighted by a black or red spot. Bilateral pain was represented in 49 out of 132 drawings (37,1%), while unilateral pain appeared in 43 drawings (32.6%). Iconographic elements mostly associated with migraine included unilaterality, pulsating quality, worsening with physical activity, nausea, vomiting, photophobia, phonophobia, and aura. Tension-type headache was represented most frequently with bilateral pain, oppressive in quality, and stress-related.

The concordance of the artistic and clinical diagnoses for migraine and tension-type headache was 78.5% and 78.6%, respectively, when the drawings were assessed by a neuropediatrician. This concordance was similar for migraine drawings assessed by both neurologists (76.3% and 83.6%), but not for tension-type headache (35.1% and 48.4%). The agreement between neurologists was moderate and similar for both types of headaches (migraine, k: 0.51; tension-type headache, k: 0.5). Among all the drawing elements, those previously associated with migraine demonstrated a high grade of correspondence with the clinical diagnosis of migraine; particularly, presence of aura, worsening with physical activity, nausea and vomiting showed a 100% concordance [20].

Table 1 summarizes various studies that have analyzed the use of drawings as a diagnostic tool for headaches in children and adolescents. Overall, these studies highlight that drawings can be an effective method for understanding pain perception in young patients and supporting clinical diagnosis.

The studies involved groups of children and adolescents aged between 4 and 19 years, who were asked to create drawings to gather information about pain localization, intensity, and symbolic characteristics. Analyses, often conducted by neurologists or neuropsychiatrists, revealed recurring elements such as:

- High diagnostic sensitivity: the study by Stafstrom et al. [11] reported a sensitivity of 93% and a specificity of 83% in using drawings for headache diagnosis.
- Symbolic and chromatic elements: Drawings frequently included elements such as lightning bolts, tears, and dark colors associated with migraines. For instance, Mosquera et al. (2008) [17] observed that 75% of children with migraines depicted unilateral pain, while 60% used symbolic elements like lightning bolts or tears. Similarly, Wojaczynska-Stanek et al. (2008) [16] found that 73% of children with headaches used dark colors, suggesting a correlation between color tone and pain intensity.

The studies vary in terms of evidence level, with some presenting highly reliable results [15,17,18] and others based on smaller sample sizes and offering more limited evidence [19]. However, overall, research suggests that drawings can be a valuable tool to support clinical diagnosis, especially in children who may struggle to verbally describe their pain.

Despite some limitations, such as variability in interpreting the drawings and differences in analysis protocols, the results suggest that using drawings in the diagnosis of headaches in children and adolescents represents a promising approach. It can complement traditional clinical assessments and enhance the understanding of pain experiences in young patients.

In conclusion, although the number of available studies is limited, they all agree on the usefulness of drawing for differentiating between types of primary headaches and for better characterizing headache phenotypes, particularly in younger children.

However, there are several limitations that limit the generalization of data, such as the usually small sample size, the variability of age examined, not always present control sample, almost always a non-double-blind assessment, and intercultural differences in assessing symbolic aspects.

### 2.2. Drawing Use in the Adult Population

The use of the drawing has also been investigated in the adult population as a useful tool to assess the severity of headache as well as for making an appropriate differential diagnosis between headache, cluster headache, and migraine, in the absence of specific biomarkers.

Buture et al. [21] tested 150 healthy participants with 6 images portraying people with pain, asking them to rate the pictures as mild, moderate, severe, or excruciating pain. During the second phase of the study, 116 participants with headaches (16 with Cluster Headache, 100 with migraine) were asked to assess the same images in the same manner. The latter were also requested to choose the image that most accurately illustrated their headache attacks. The results allowed the authors to develop a screening tool consisting of 6 images that was able to depict headache severity, even though the images did not differentiate between cluster headache and migraine.

### 2.3. The Use of Pain Charts

Although the usual focus in pain measurement is primarily pain intensity, it is also important to investigate other features such as frequency, duration, and localization of pain.

Baeyer et al. [22] explored the use of pain charts not only in the assessment of localization of pain but also for examining other aspects such as severity and sensory quality, recurrent or chronic pain, using symbols, shading, or colors. One of the main issues in using pain charts within multidimensional pediatric pain questionnaires and diaries is that many children below the age of 8 years often require adult assistance to complete them, which may bias their responses. Another challenge involves determining the number, size of different locations or areas of pain that need to be differentiated. On the other hand, in clinical settings, pain charts provide significant flexibility. They allow the integration of color and symbolic representations to illustrate not just the presence or absence of pain but also its quality and intensity. These visual tools, however, must be developed collaboratively between individual patients and clinicians to ensure accuracy and relevance. Nevertheless, pain charts may not always be the most effective method for determining pain location. In certain cases, particularly when only a few areas are of concern, checklists focusing on specific body regions may be more appropriate.

Over the next decade, the authors [22] expect to see a knowledge transfer and standardization, as seen in the widespread adoption of the 0–10 metric in face, in visual analog and numerical scales for evaluation of pain intensity. A similar evolution is expected in tools for assessing pain location and quality.

### 2.4. Children’s Pain Perception and Expression

Children identify pain as originating from three primary sources: external, internal, and emotional. External pain is often visible and associated with injuries like cuts or burns, making it more comprehensible to children [23]. Internal pain, associated with diseases, is harder for children to understand as they struggle to grasp its cause and implications [24]. Emotional pain, often described as “pain in the heart”, is linked to psychological distress from events such as losing a loved one [25,26].

In children’s perception, pain is closely associated with physical harm or physical discomfort [23]. Drawings and verbal descriptions frequently depict pain through bodily injuries, often using colors like red and black to represent its severity. This association is particularly strong with external sources of pain, but can also be linked to internal conditions. Pain is universally regarded by children as an unpleasant experience characterized by sadness, discomfort, and distress [26]. The intensity of pain ranges from mild discomfort to extreme suffering, at times perceived as life-threatening [23]. Pain is also linked to both physical sensations (such as heat or pressure) and emotional distress (such as fear or anxiety). It is often perceived as a sign of danger or illness, leading to anxiety in children [23,25,27,28].

They associate pain with potential long-term consequences, such as restrictions on daily activities, and with medical interventions (such as injections). Some children interpret pain as a form of punishment, reinforcing feelings of guilt or distress [29]. Experiences of Hospitalization and medical procedures can further shape their understanding of pain, often turning it into a source of fear and apprehension. Children’s descriptions of pain are influenced by their personal experiences. Healthy children tend to focus more on the physical aspects of pain, while chronically ill children are more likely to emphasize its emotional and psychological effects. Acute pain is often seen as severe but temporary, whereas chronic pain is associated with prolonged suffering and frustration. The colors red and black are commonly used to depict pain in drawings, representing distress and uncertainty [26]. In some cases, children express their pain in self-centered ways, describing personal experiences rather than generalizing their understanding [30,31,32,33].

## 3. Correlation Between the Clinical Phenotypes and the Graphic Representation of Pain

According to IHS Classification-ICHD-3, migraine and tension-type headaches differ significantly in their clinical presentation. Migraines are more often unilateral, with a pulsating quality of pain, a moderate or marked pain intensity, and aggravation by or causing avoidance of routine physical activity, and are associated with nausea and/or vomiting or photophobia and phonophobia.

On the other hand, the pain in tension-type headache is more often bilateral location, with a pressing or tightening (non-pulsating) quality, mild or moderate intensity, not aggravated by routine physical activity such as walking or climbing stairs and not associated with both nausea or vomiting and no more than one of photophobia or phonophobia.

In children and adolescents, migraine headache is more often bilateral than is the case in adults; unilateral pain usually emerges in late adolescence or early adult life. Migraine headache is usually frontotemporal. Occipital headache in children is rare and calls for diagnostic caution [3]. A subset of otherwise typical patients have facial location of pain, which is called “facial migraine” in the literature [34]; there is no evidence that these patients form a separate subgroup of migraine patients. In young children, photophobia and phonophobia may be inferred from their behavior [3].

Considering the clinical differences between headaches in adults and those in children, effective diagnostic tools have recently been developed to support the diagnosis of headaches in individuals under 18 years of age. ID Migraine is an easy and quick-to-administer questionnaire that serves as a reliable screening and early diagnosis, already used in adults, which has recently been validated in an Italian version for children over the age of 6 and adolescents [35].

All these instruments allow pediatric neurologists to make a clinical diagnosis together with a complete medical history and a neurological physical examination. Alongside the various tools available for clinical diagnosis, the use of artistic diagnosis emerges as a complementary approach.

We define the artistic diagnosis as a diagnosis made following the evaluation of patient drawings by neuropediatricians and neurologists.

An accurate artistic diagnosis can be supported by the identification of specific, easily detectable graphic elements in children’s drawings.

In terms of localization, pain can be differentiated into unilateral and bilateral.

In cases of unilateral pain, representations may include a hand placed on one side of the head, written descriptions explaining where the pain is felt, circles or arrows designating the exact area of discomfort, objects or lines positioned on one side of the head, or sparkles appearing on that side. For bilateral pain, images may show both hands covering each side of the head, circles or arrows indicating both painful areas, or bandages and circles around the head [11,14,15,16,17,18,19,20].

Different types of pain quality are also represented with specific symbols. Pulsating pain might be depicted using visuals like hearts, hammers, drums, pendulums, or with onomatopoeic words like “pum pum” or “bam bam.” On the other hand, pressing pain could be symbolized by images of stones or weights placed on the head, clamps, hands gripping the head, or gears. Pain that creates a sensation of tightness or constriction is often portrayed with bandages or knots squeezing the head. Stabbing pain is frequently illustrated with knives or drills, while burning pain is commonly represented with flames, candles, or fire [11,14,15].

The intensity of pain is also shown through various graphic cues. Lower intensity pain might be represented with smaller drawings, pain located outside the head, bright and light images, and neutral or smiling facial expressions. Higher intensity pain, in contrast, could be depicted with larger illustrations, pain centered inside the head, darker images, the use of red or black, expressions of sadness or distress, tears, and a person lying down. Scripts or onomatopoeia may be added to further describe the intensity [12,15,16].

Other symptoms, such as the worsening of pain with physical activity or the urge to avoid it, can be visually shown by images of a person lying in bed or written descriptions explaining the avoidance of movement. Nausea is often symbolized by hands on the abdomen or mouth, while vomiting can be represented by visuals of a toilet, vomit, or hands clutching the stomach [11,14,16,17,18].

Sensitivity to light is often depicted by crossed-out light sources such as the sun or lamps, along with scripts describing the discomfort caused by light exposure. Phonophobia may be represented by crossed-out noise sources like voices, drums, musical notes, or horns, accompanied by scripts explaining the distress caused by sounds [11,14,15,16,17,18,19,20].

Lastly, visual auras can be represented with images of stars, colored spots, zigzag lines, half-objects or faces (hemianopsia -half-field visual impairment), or fog. Other types of auras, like sensory, speech, language, and motor, may not have specific visual representations, but brainstem auras can include symbols such as double vision, fog, lines or circles around the head, or spirals [11,18].

We propose a comprehensive list of iconographic elements of typical headache symptoms that are easily recognizable in the drawings of patients and control subjects. For the main characteristics, we also report the positive predictive value for migraine reported in the literature (see unilateral lateralization) (Table 2).

### 3.1. Migraine with Aura in Children

The International Classification of Headache Disorders, 3rd edition (ICHD-3) defines aura as a group of neurological symptoms that last between 5 and 60 min, typically preceding or occurring within an hour of a migraine headache. A diagnosis of migraine with aura (MA) requires a minimum of two episodes with completely reversible neurological symptoms, which may include visual, sensory, language-related, motor, brainstem, or retinal disturbances. Additionally, at least three of the following characteristics must be present:

- Gradual appearance of a symptom over five minutes or more;
- At least two symptoms occurring sequentially;
- Each symptom lasts between 5 and 60 min;
- At least one aura symptom is unilateral;
- At least one positive symptom (e.g., flickering lights, tingling);
- The aura is followed by or coincides with a headache within one hour.

These diagnostic criteria are applicable to both adults and children. ICHD-3 further categorizes migraine with aura into typical aura, brainstem aura, hemiplegic migraine (familial or sporadic), and retinal migraine. The hemiplegic and brainstem aura subtypes are considered more severe and can influence treatment decisions [10,37].

Approximately one-third of children and adolescents with migraines experience an aura phase [38]. The most commonly reported aura in pediatric cases is visual aura, followed by somatosensory and language-related symptoms [38].

Visual disturbances may manifest as:

- Blurred vision;
- Zigzag lines;
- Scotomata (partial vision loss);
- Scintillations (flashes of light);
- Black dots;
- Kaleidoscopic patterns;
- Size perception distortions (micropsia/macropsia).

Other forms of aura include numbness or tingling (sensory disturbances), speech/language difficulties (aphasia, dysarthria), motor impairments (hemiparesis), and brainstem-related symptoms such as vertigo, tinnitus, and double vision [39].

Aura symptoms can differ between episodes and may be challenging for young children to describe verbally. In clinical practice, children’s drawings are often utilized to communicate visual disturbances and differentiate migraines from other headache disorders [40].

### 3.2. Neurological Mechanisms of Aura

The physiological foundation of aura is believed to involve cortical spreading depression (CSD), a wave of depolarization followed by hyperpolarization that slowly moves across the cerebral cortex [41].

As CSD spreads over particular brain regions, temporary neurological symptoms emerge and then subside. This process disrupts ionic balance, neurotransmitter function, and blood circulation in the brain, all of which play a role in migraine pathophysiology [42].

### 3.3. Differential Diagnosis Between Visual Hallucinations in Migraine with Aura and Epilepsy

Migraine and epilepsy share several clinical features, including episodic attacks with paroxysmal onset and distinct preictal, ictal, and post-ictal phases, characterized by changes in mood, behavior, consciousness, and sensory or motor functions. This overlap suggests common underlying mechanisms of neuronal hyperexcitability, modulated by subcortical pathways. Despite some well-established differences in clinical presentation, differential diagnosis can be challenging, particularly with atypical cases. Cortical spreading depression (CSD) is thought to be the mechanism behind aura in migraine and visual symptoms in occipital epilepsies.

Differentiating occipital lobe epilepsy from migraine can be challenging due to symptoms overlap, particularly simple visual hallucinations. Accurate diagnosis requires careful evaluation of the timing and characteristics of visual disturbances, especially in pediatric cases, where both conditions may co-occur. Epileptic ictal visual hallucinations are characterized by distinct attributes, including color, shape, size, movement, and duration.

The most reliable indicators of migraine visual aura are its accompanying symptoms, such as nausea, vomiting, photophobia, phonophobia, and prolonged headache. A duration of more than 5 min strongly favors migraine aura (median duration of 20 min) over epilepsy aura (median duration of 56 s), with high diagnostic accuracy. However, some children with migraine may experience shorter or atypical auras, and in rare cases, visual aura can occur without headache, making diagnosis more complex. Common visual symptoms in children include black-and-white zigzag patterns, peripheral visual disturbances, scintillating scotomas, and blurry vision. Less frequently, unusual colors, brightness, micropsia, teleopsia, and short-lasting visual snow may be present. Rarely, conditions such as palinopsia, visual snow syndrome, Alice in Wonderland syndrome, or complex visual hallucinations suggest alternative diagnoses. In this framework, drawing interpretation may significantly aid differential diagnosis by facilitating the identification of pathognomonic aura characteristics. For further information, we refer the reader to a review that fully addresses this issue [43] and in supplementary files we enclose a Table S1 reporting the differential diagnosis of visual symptoms between migraine and epilepsy.

### 3.4. Migraine Triggers in Children

Patients with migraines often report identifiable triggers—internal or external stimuli that increase the likelihood of an attack. These “trigger factors” can be categorized into behavioral, environmental, infectious, dietary, chemical, and hormonal factors, highlighting the hyperexcitable nature of migraine brain and its heightened reactivity to various stimuli. Identifying and avoiding these triggers is essential for managing migraines effectively. Among pediatric patients, the most commonly reported migraine triggers include sleep deprivation, stress, warm climate, noise, and bright lights; stress is the most significant trigger, particularly stress from home and school environments. In children, the onset of migraines typically occurs within three hours of trigger exposure. Dietary factors also play a crucial role, with foods like chocolate, caffeine, milk, and cheese being frequently associated with migraine episodes. The impact of diet on migraines may involve alterations in serotonin and norepinephrine levels, changes in blood vessel function, or activation of critical brain pathways, further emphasizing the role of nutrition in migraine management for children and adolescents [44].

## 4. Future Directions

A standardized protocol should certainly be considered to improve the use of drawing in the diagnosis and management of childhood headaches, including, for example, introducing the drawing before the meeting with the doctor so as not to influence the child, or asking school children to accompany the drawing with a free written description. The use of artificial intelligence and machine learning algorithms presents a promising way to analyze large datasets of children’s drawings. These technologies may reveal patterns, similarities, and diagnostic indicators that are not readily apparent through clinical observation alone. Such tools could help establish an independent, standardized assessment grid. However, this would require the digitization of a substantial volume of drawings. Adolescents would probably use artificial intelligence applications more favorably than drawing on paper more suitable for younger ages. It will also be necessary to provide clinical training for the interpretation of these drawings if this tool is to be effectively integrated into common clinical practice. Finally, it can be a useful tool for evaluating the effectiveness of management, especially in minors with anxiety problems and introversion.

Finally, interesting insights may arise from post-potential biomarker studies in pediatric headache [45].

## 5. Gallery

The review of the literature, although with limitations and variability among the different authors, supports the usefulness of drawing as a tool for the diagnosis and management of children with headaches. Here we present a gallery of drawings by our children, expanding the collection of drawings already reported in the literature [12,15,16,17,18,19,20,21,36]. We made an effort to identify and classify correlations between graphic representations and the clinical phenotypes of pediatric headache. These illustrations have been analyzed to classify iconographic features associated with the core characteristics of migraine—with and without aura—as well as tension-type headache. The visual elements observed are largely consistent with the existing literature, although several novel and clinically relevant representational patterns have emerged. Further in Supplementary Figure S1 File we added a Glossary reporting figurative symbols, based on our drawings, and their clinical meanings.

**Figure 1:**
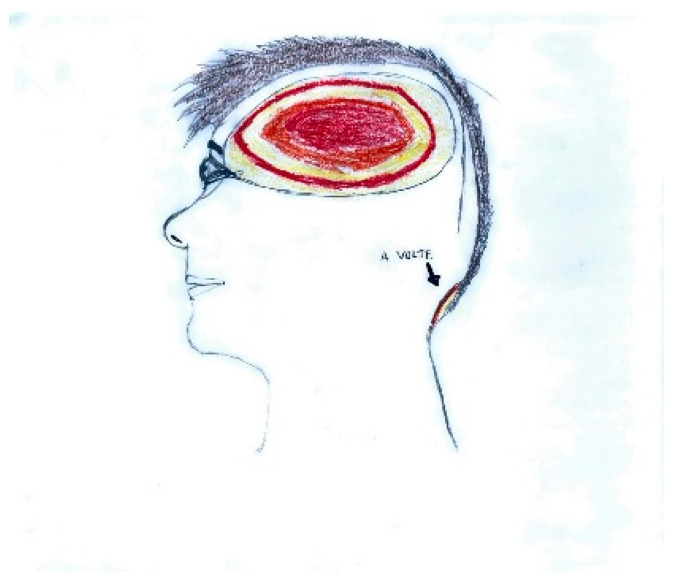
Male, 12 years old. Migraine without aura. Red color is focus of most intense pain. With the written word the child refers to complain pain “sometimes” in the occipital region.

Pain localization is frequently depicted through visual markers such as circles, directional arrows, or shaded regions. As shown in Figure 1, the parieto-temporal area is delineated with a red outline, highlighting the central focus of intense pain, while adjacent areas are shaded in lighter tones to indicate lesser involvement. The inclusion of an arrow pointing toward the occipital region, accompanied by the word “sometimes,” reflects the intermittent nature of pain in that area.

**Figure 2:**
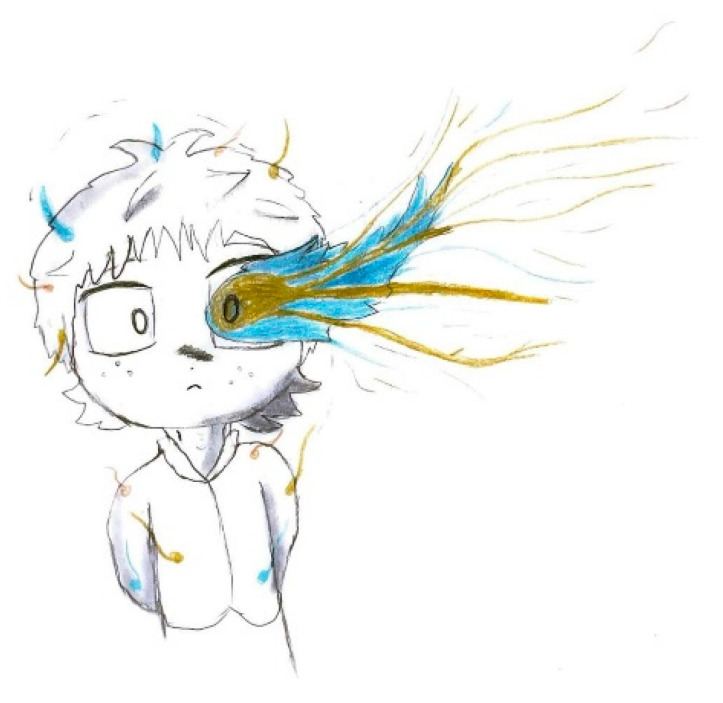
Male, 12 years old. Migraine. Unilateral orbital pain with bursts of color and flame-like patterns.

In Figure 2, retro-orbital pain is rendered through bursts of color and flame-like patterns emanating from the eye, effectively conveying the burning and explosive quality of the discomfort.

**Figure 3:**
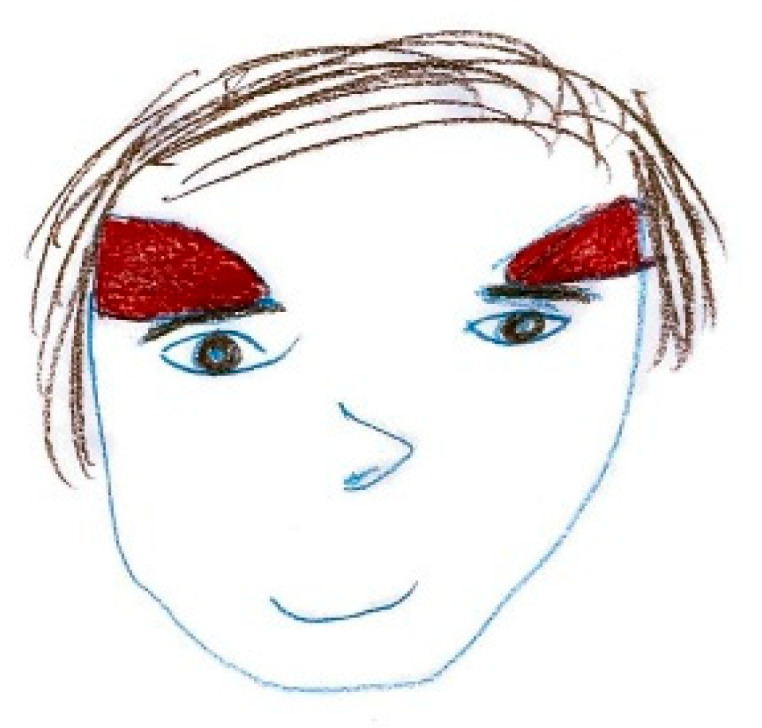
Male, 14 years old. Chronic migraine. Bitemporal area of pain.

**Figure 4:**
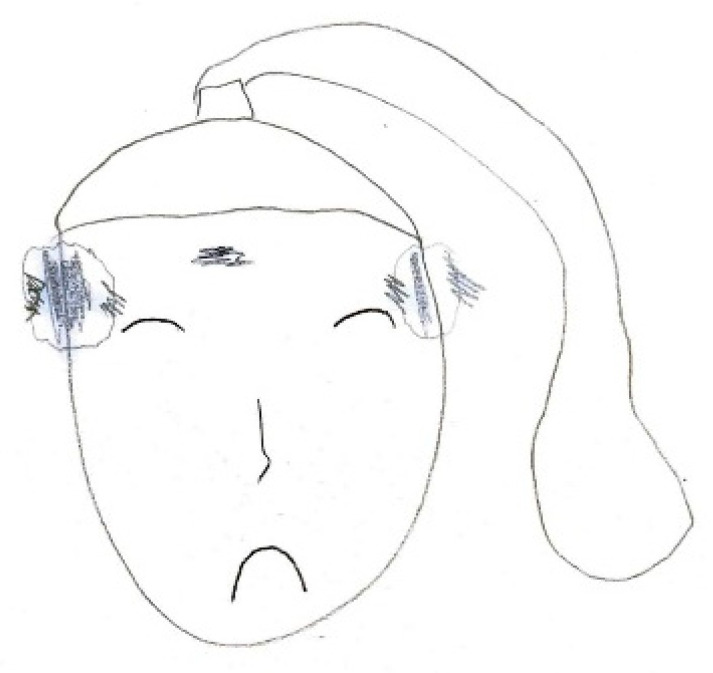
Female, 10 years old. Bilateral pain. Severe distress.

Figure 3 illustrates localized, shaded areas in the temporal and frontal regions, accompanied by a facial expression indicative of significant distress, highlighting the severity of the pain. In contrast, Figure 4 exemplifies bilateral pain presentation, with symmetrical red markings on both sides of the head.

**Figure 5:**
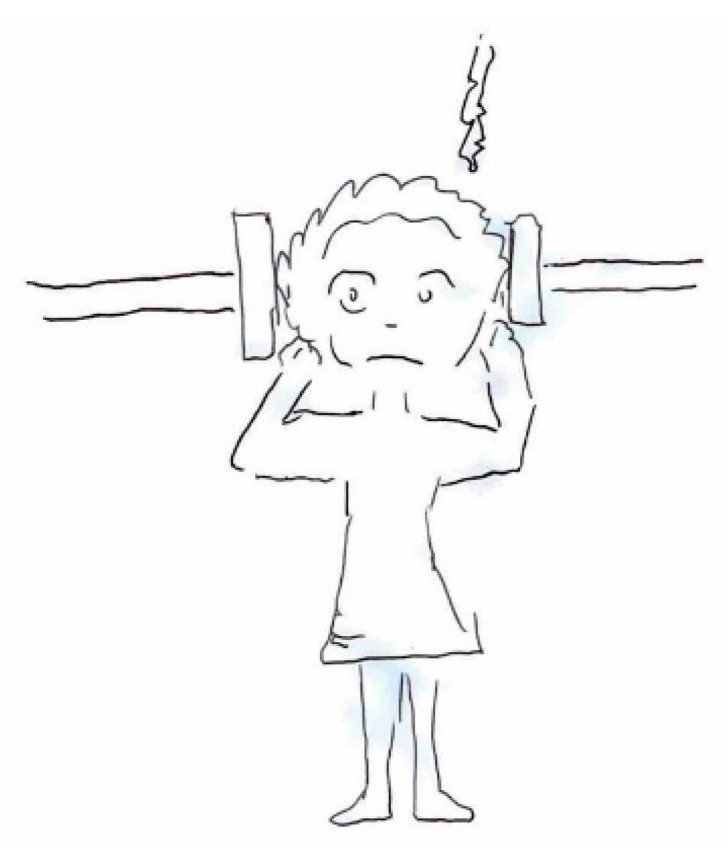
Female, 11 years old. Tension headache. Pulling pain.

**Figure 6:**
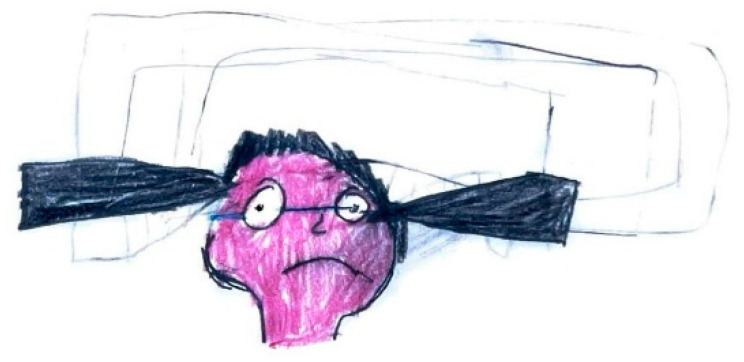
Male, 10 years old. Migraine. Stabbing headache.

The quality of pain is often symbolized by various objects offering insights into its distinctive characteristics. For instance, pressing pain is typically represented by an object constricting the head, such as a press (Figure 5), while stabbing pain is often suggested using sharp objects such as knives or spikes (Figure 6).

**Figure 7:**
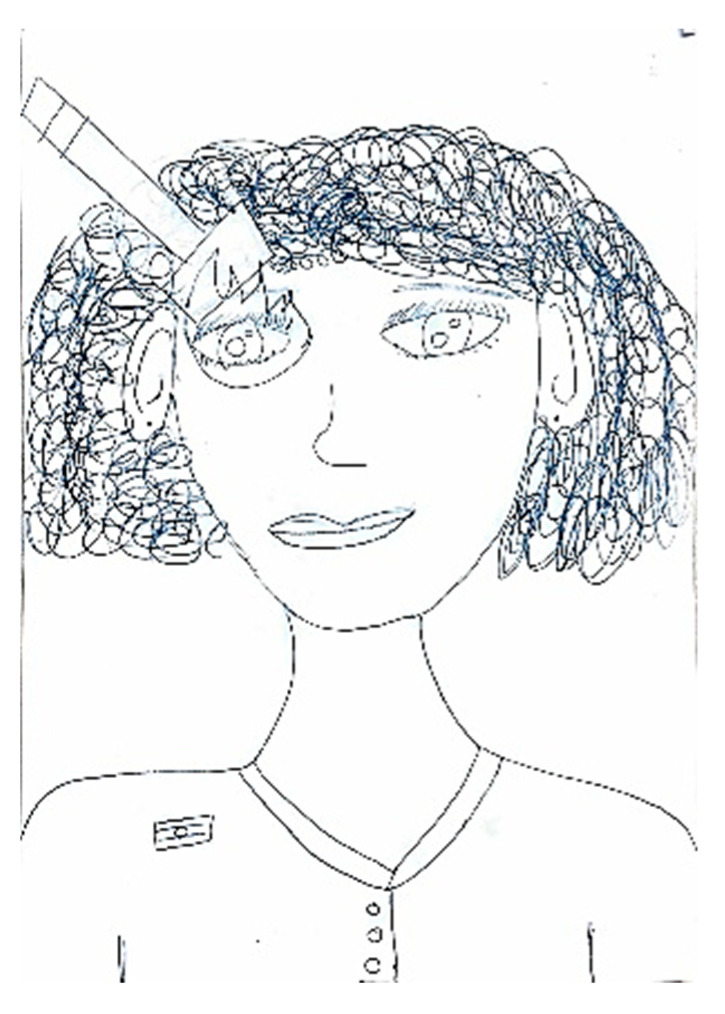
Female, 12 years old. Migraine. Unilateral pulsating pain in supraorbital area.

**Figure 8:**
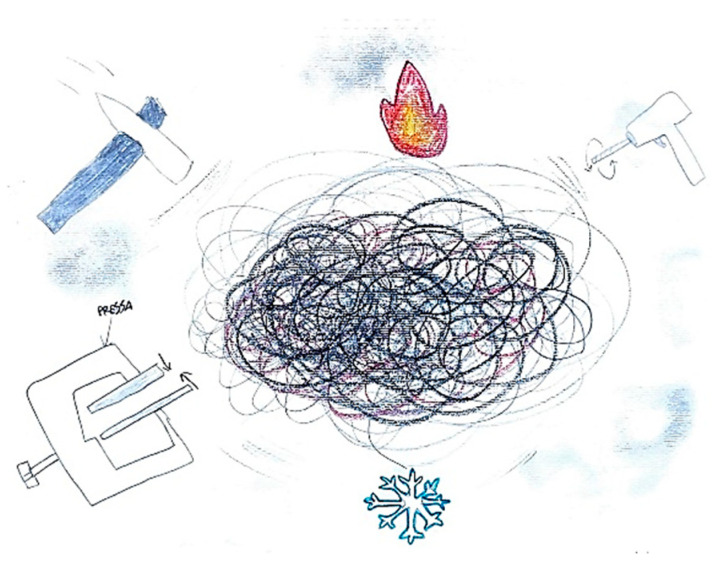
Male, 11 years old. Migraine. The child describes different qualities of pain: hammering, burning, stabbing, freezing, or pressuring. Brain fog. The black written word is: Press.

It is not uncommon for pediatric patients to simultaneously perceive and depict multiple pain qualities within a single illustration. For example, in Figure 7, the image of a hammer striking the temporal region symbolizes the pulsating nature of the headache, while the presence of a flame in the ocular area conveys the burning sensation characteristic of retro-orbital pain. Similarly, Figure 8 illustrates the coexistence of diverse pain modalities: a hammer indicating pulsating pain, a drill representing stabbing sensations, and a press suggesting a compressive or pressing quality. These elements are further enriched by visual cues associated with thermal sensations, such as representations of heat and cold. Collectively, these illustrations underscore the multidimensional and complex nature of pain perception in children, highlighting the utility of drawing as a nuanced diagnostic tool.

### 5.1. Intensity of Pain

**Figure 9:**
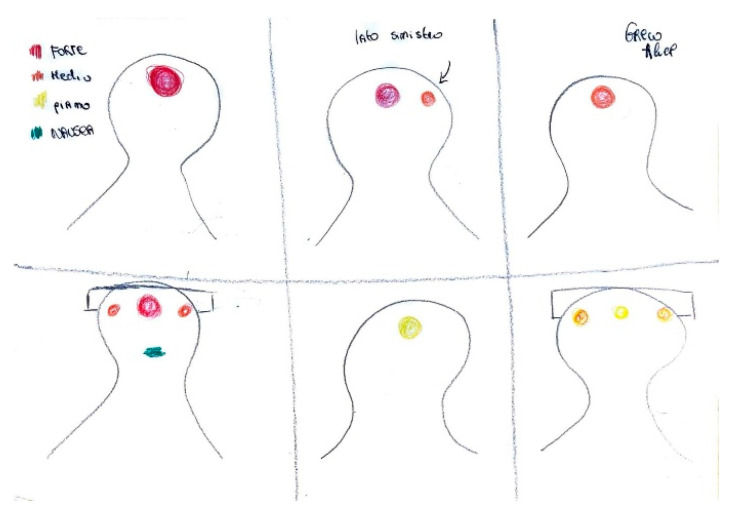
Female, 10 years old. Migraines and tension headache attacks. The girl describes intense pain in red = severe; orange = moderate; yellow = mild, and green = nausea.

In children, the numerical concept related to pain intensity scales is often not yet acquired. Additionally, rather than quantifying pain intensity using adjectives, children prefer to express it through color scales and by representing the degree of associated disability Figure 9.

It is also possible to observe that pain representations outside the head are more frequently associated with lower pain intensity, whereas higher intensity is more often depicted inside the head.

**Figure 10:**
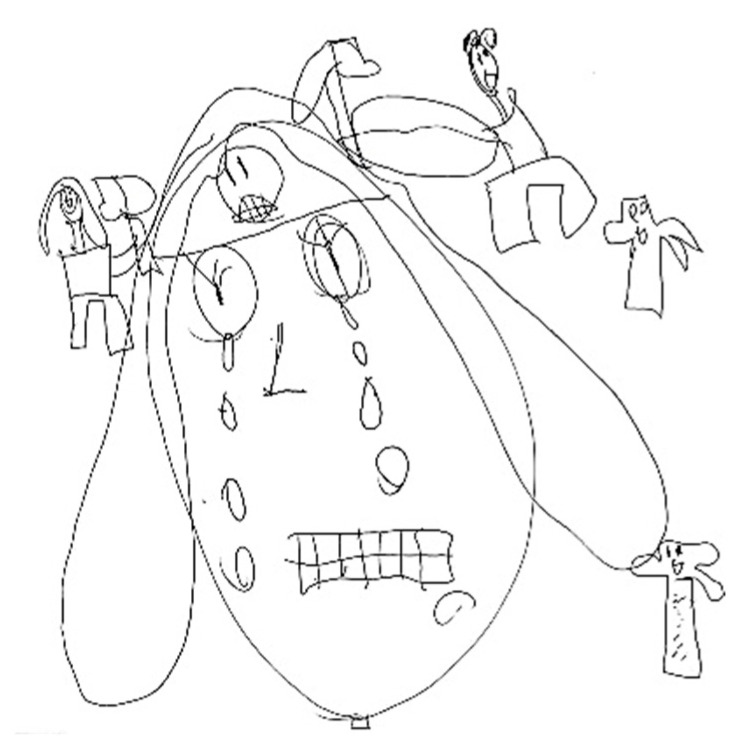
Female, 8 years old. Migraine. Severe pain with lacrimation. Little men hammer the head.

**Figure 11:**
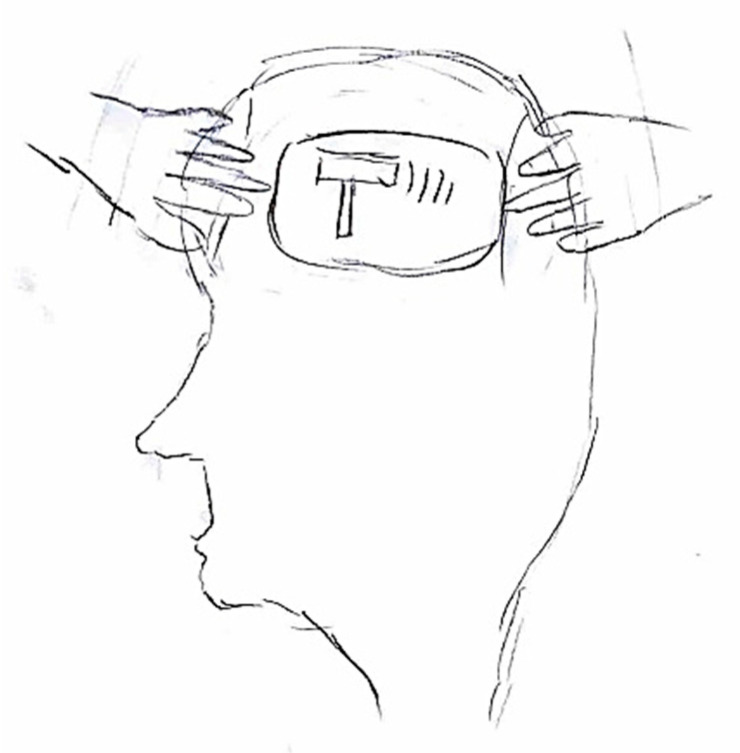
Female, 12 years old. Migraine. Mixed quality of pain: pounding and pulling. Moderate intensity.

Colors such as red and black are preferentially chosen to represent very strong pain, and severe distress is often reflected in depictions of faces with sad expressions, frequently accompanied by tears. Figure 10 and Figure 11.

### 5.2. Related Symptoms and Behaviors

**Figure 12:**
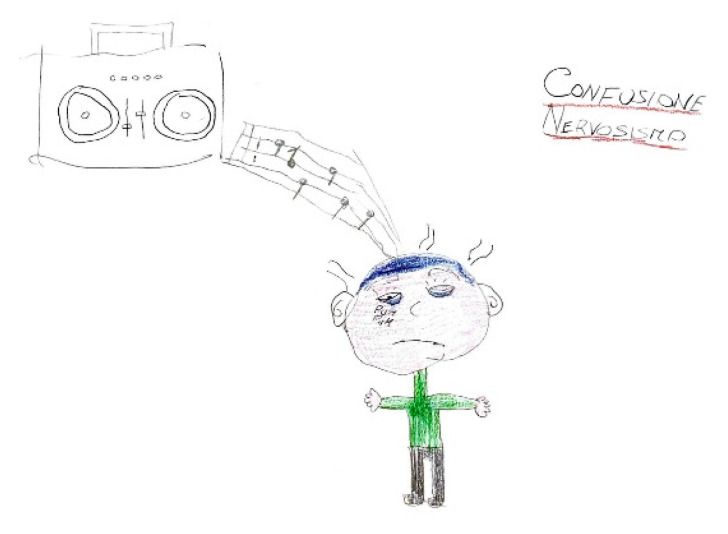
Male, 13 years old. Migraine. Sonophobia. The written words are: Confusion, Nervousness.

**Figure 13:**
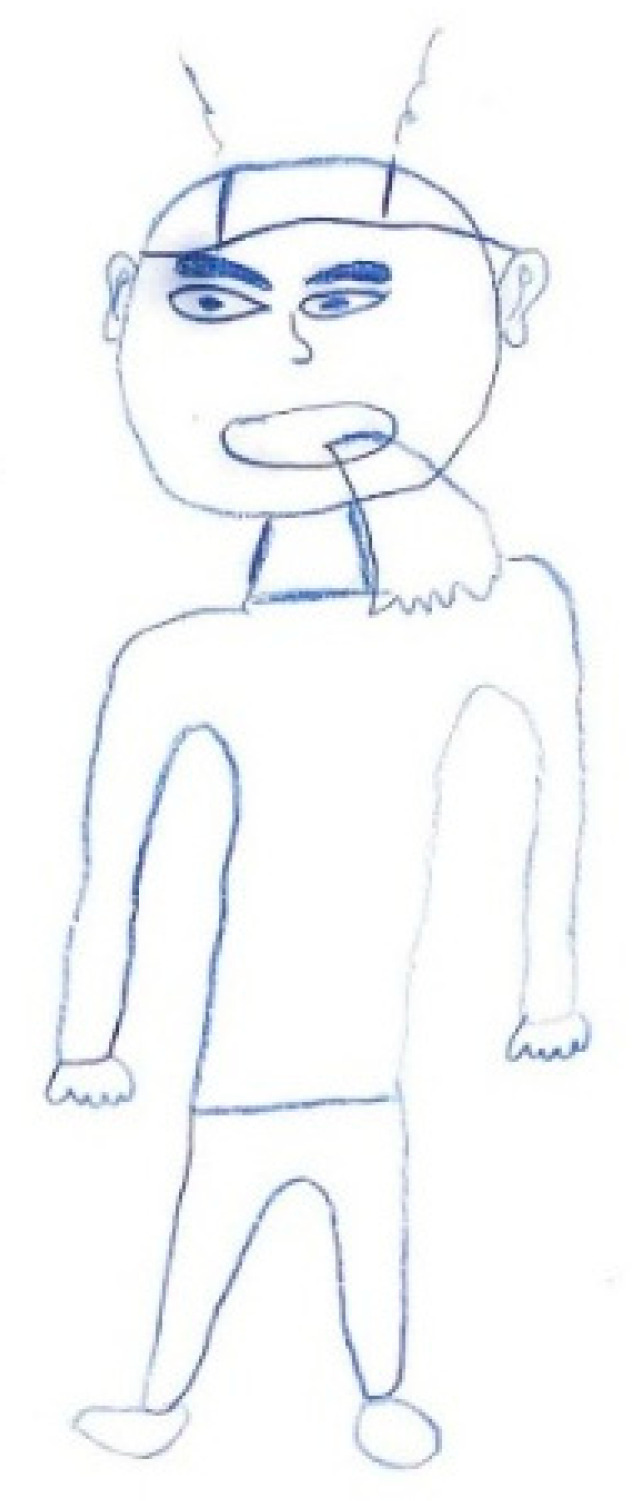
Male, 10 years old. Migraine. Vomit.

**Figure 14:**
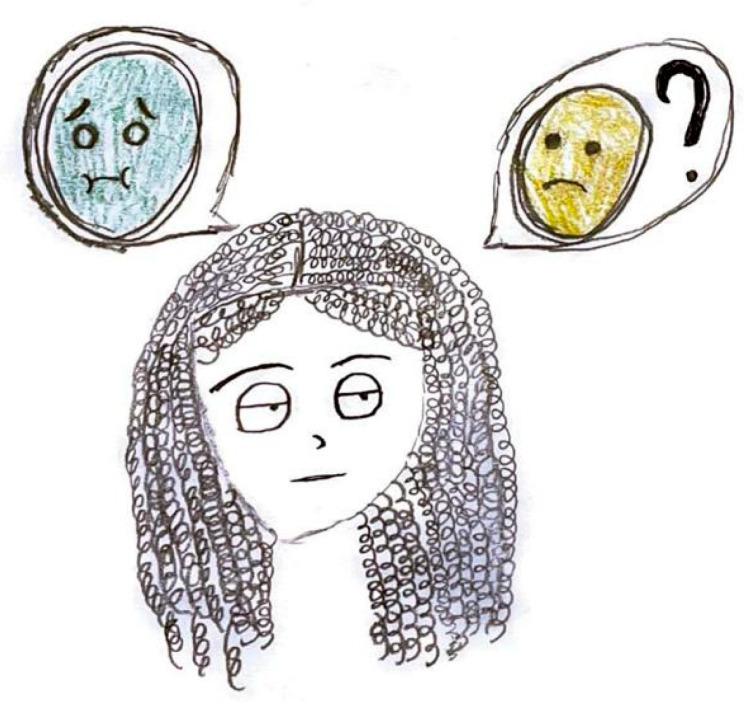
Female, 11 years old. Migraine. In the circles, the light blue color indicates vomiting, and yellow indicates nausea.

**Figure 15:**
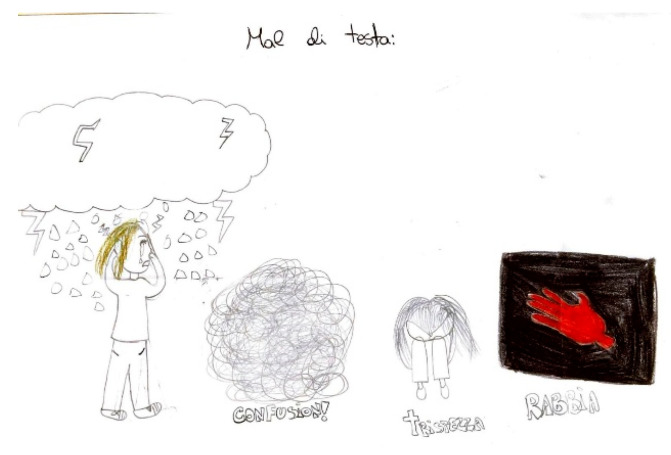
Female, 11 years old. Migraine. The drawing shows confusion (2° drawing to the right), sadness and anger. The written word are Headache in the top of the figure, Confusion in the second drawing, sadness in the third drawing and Anger in the fourthy drawing.

A headache is frequently accompanied by a range of symptoms and behavioral changes, including confusion and mood alterations. Classical manifestations typically include phonophobia and photophobia, which patients often depict using symbolic representations of light or sound. For instance, in Figure 12, auditory stimuli are symbolized by musical notes that induce discomfort, emphasized by distressed facial expressions and onomatopoeic words like “pum-pum,” underscoring the pulsatile and intense nature of the pain. Nausea and vomiting are common features associated with migraines, often clearly depicted, such as in Figure 13, or evoked through blurred colors or shades of green and yellow to represent physical illness. Mental confusion, which can occur before, during, or after a migraine episode, is also commonly portrayed. In Figure 14, nausea is conveyed through an expression of disgust, while confusion is symbolized by a bewildered expression and a question mark. Figure 15 illustrates the “brain fog” phenomenon, which is a feeling of difficulty concentrating, mental confusion, and cognitive fatigue, represented by a cloud, alongside the emotional impact of the headache itself, which becomes a source of sadness and anger.

**Figure 16:**
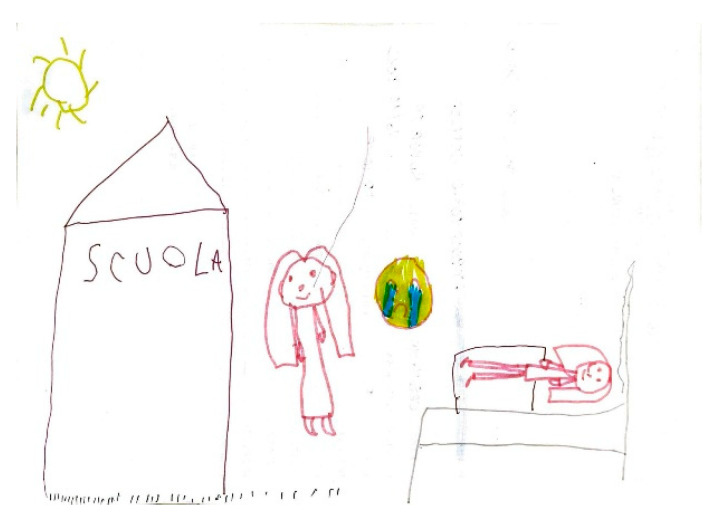
Female, 7 years old. Migraine. Girl lost the school when has a painful attack and had to lie down.

**Figure 17:**
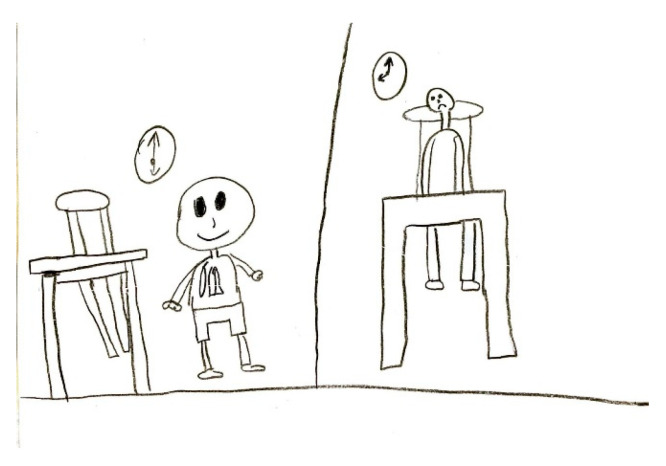
Male, 9 years old. Migraine. No pain/pain.

Occasionally, the discomfort linked to a headache manifests as a change in the child’s behavior, such as retreating to bed or being unable to engage in regular daily activities, including scholastic tasks (Figure 16 and Figure 17).

### 5.3. Aura

**Figure 18:**
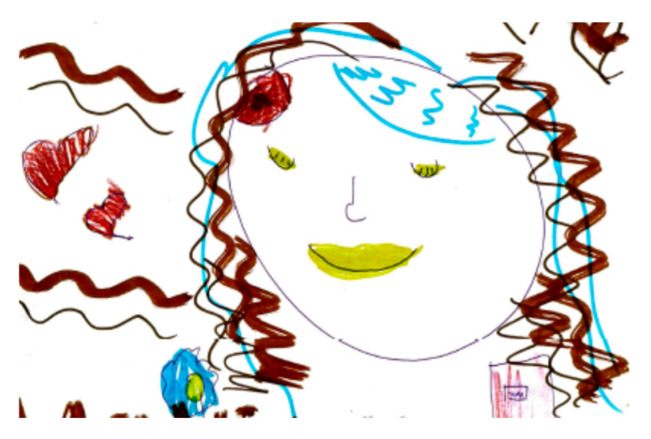
Female, 8 years old. Migraine with aura: Unilateral left pain. Visual aura: zigzag lines.

**Figure 19:**
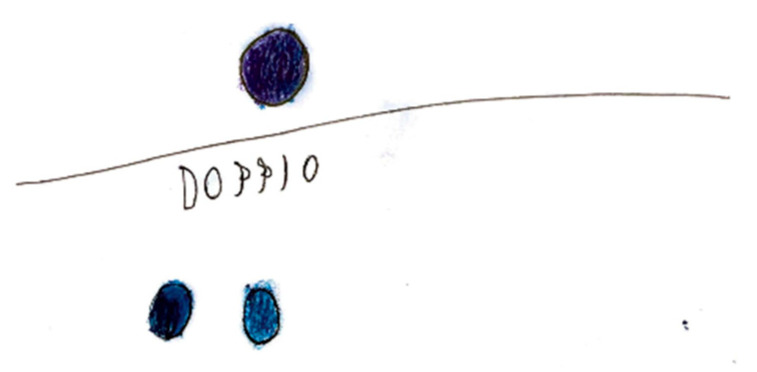
Female, 9 years old. Migraine with aura. Girl draws double vision.

Migraine aura is often represented visually as stars, dots, zigzag lines, vision loss, or fog Figure 18. Hemianopia is depicted with half-figures, such as a television screen half-obscured. Diplopia is characterized with a doubling of observed figures Figure 19.

**Figure 20:**
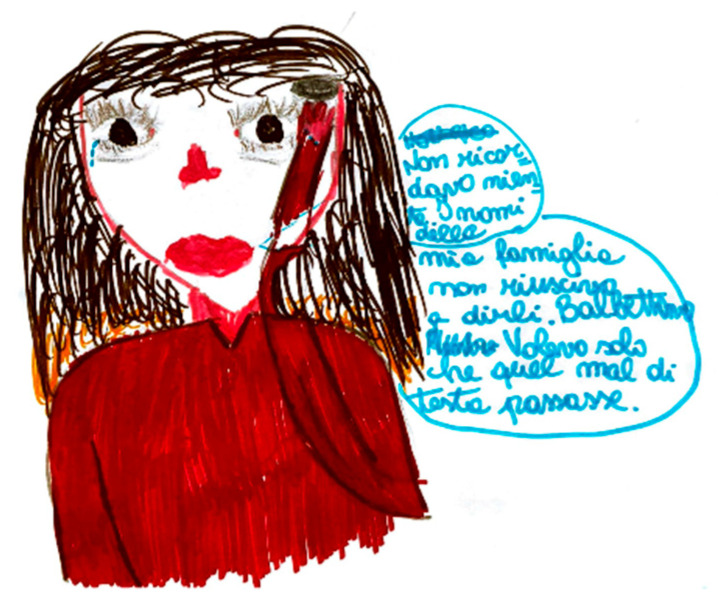
Female, 9 years old. Migraine with language disorders. The girl says, “’I couldn’t say the names of my family members, I stuttered” and complains of memory troubles.

In one of the analyzed drawings, aphasia is represented with an explanatory speech bubble: “I couldn’t say the names of my family members, I stuttered.” Figure 20.

### 5.4. Alleviating Factors and Therapies

**Figure 21:**
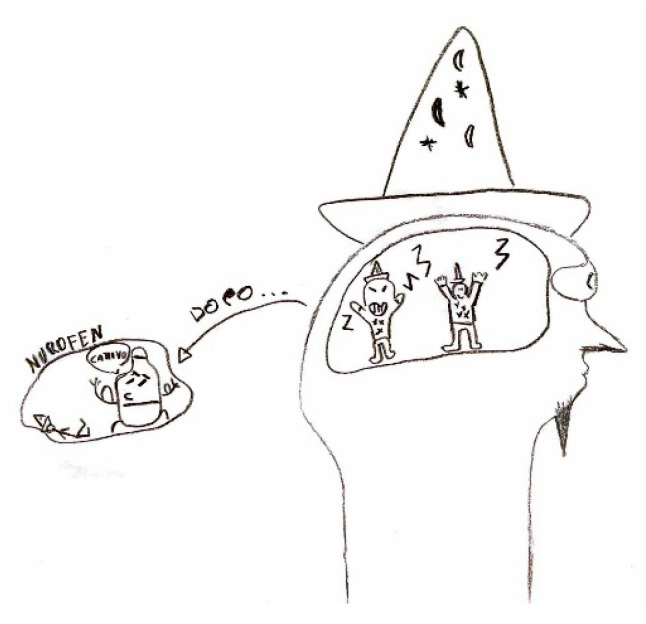
Male, 11 years old. Migraine. Aliens that bring pain then are killed by the drug (ibuprofen). The written words are: “after “ (after taken the symptomatic analgesic) and Nurofen (Ibuprophen) and in the balloon the drug says “Bad” to the painful man killed by it.

**Figure 22:**
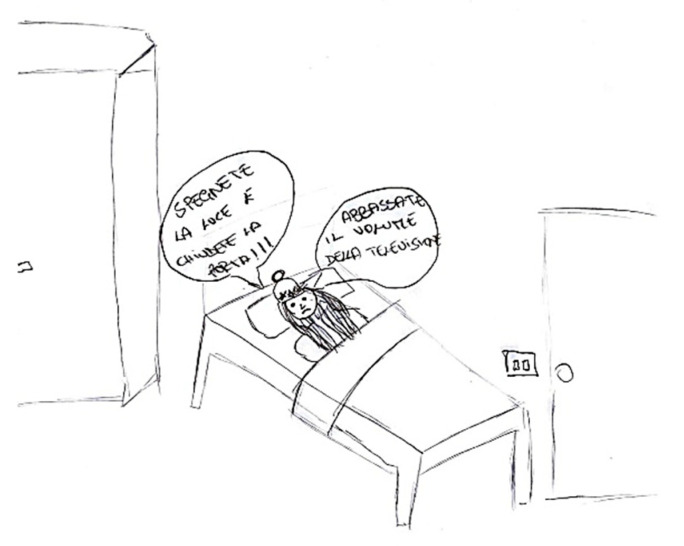
Female, 14 years old. Migraine. The girl writes turn off the lights (photophobia) and turn down the noise (sonophobia). In the first balloon the child says “Turn off the light and close the door” and in the second balloon says “turn down the volume of the television”.

In some cases, the focus of the graphic representation shifts toward certain pain-relieving approaches, both in terms of action and behaviors, as well as pharmacological interventions Figure 21. In the first instance, habitual self-administered maneuvers include head massages or compression, along with complete rest in bed away from lights or sounds Figure 22. In the latter case, illustrating the action of medication may provide the physician with greater insight into the effectiveness of the symptomatic treatment.

### 5.5. Expression of Pain

**Figure 23:**
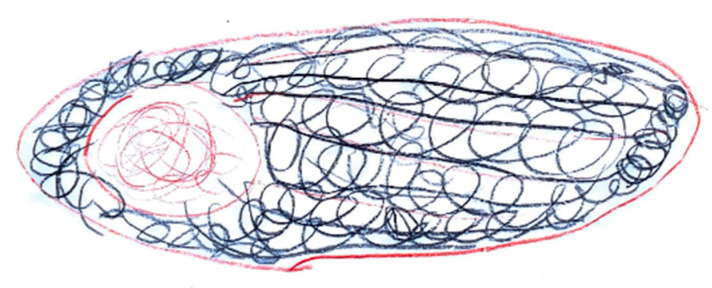
Female, 10 years old. Migraine. The drawing shows “brain fog”.

**Figure 24:**
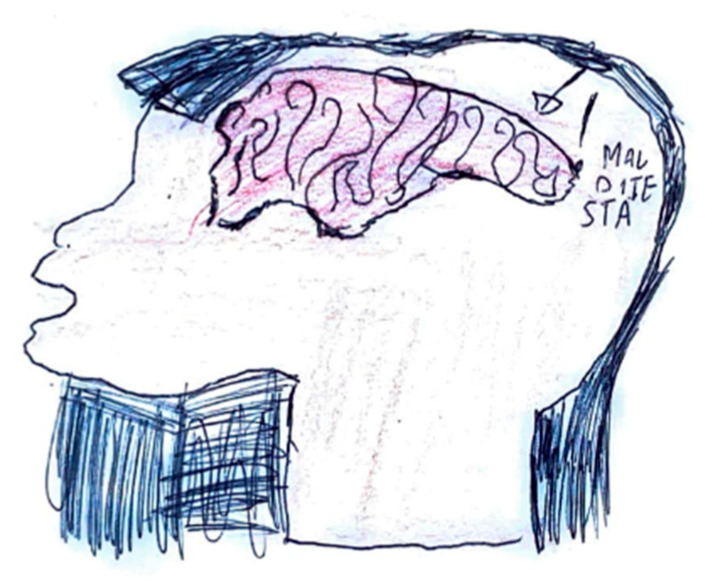
Female, 7 years old. Migraine. Question marks in the brain can represent brain fog or questions about the pain sources. The written word is: Headache.

**Figure 25:**
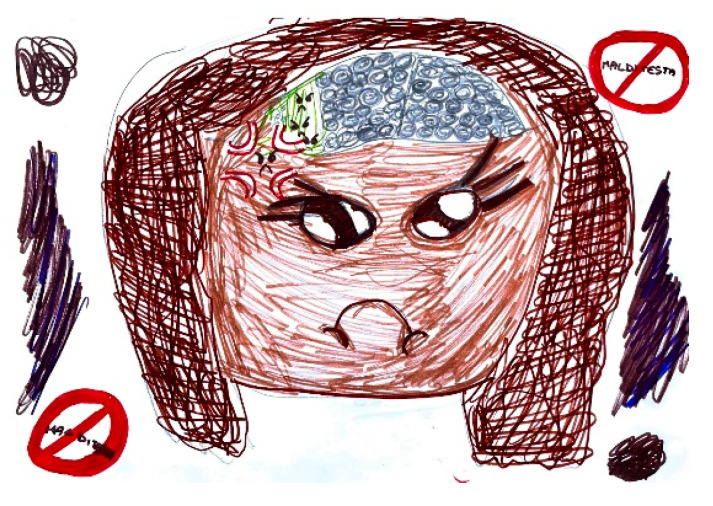
Female, 8 years old. Migraine. The drawing shows brain fog, angry face depicted with dark colors. In the stop signal the word Headache is written.

The expression of pain in children occurs in different ways, but it is possible to highlight some characteristics common to many drawings. Headaches are frequently represented as a round geometric figure, a mass, or a stone placed where the brain should be. Brain fog takes on the characteristics of a grey fog or cloud Figure 23. In children who have learned to write, spontaneous writing of captions and explanations is often associated, elaborating on how pain varies, and the emotions associated with it Figure 24. High-intensity pain is generally represented with expressions of sadness and tears Figure 25.

### 5.6. Representation of Pain in Adolescents

**Figure 26:**
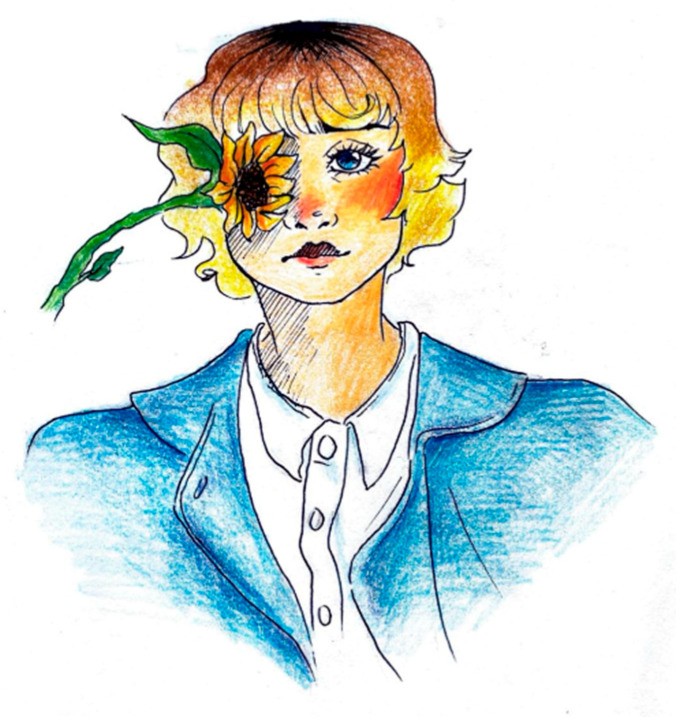
Female, 15 years old. Migraine: unilateral pain depicted in gray.

**Figure 27:**
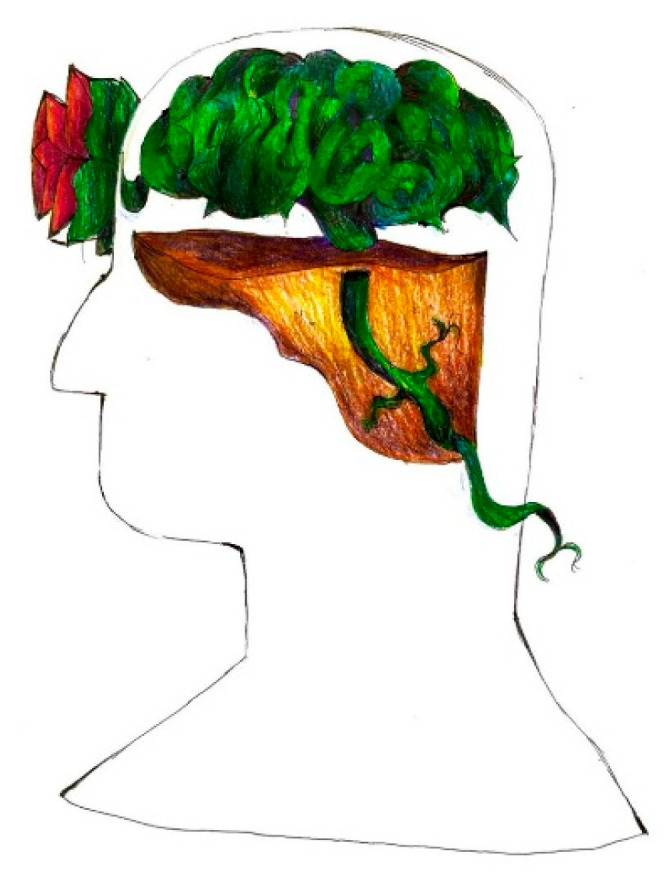
Female, 16 years old. Migraine. Pain becomes a thorny plant tightly rooted in the young patient’s head.

As descriptive and abstraction abilities mature in this population, their drawings show increasing complexity, elaboration, and often abstraction Figure 26. Rather than elementary geometric shapes, migraine pain sometimes becomes a thorny plant tightly rooted in the young patient’s head, and the areas where this manages to make its way outside the head correspond to those where the pain is perceived as better localized and more intense (for example the nape of the neck and the forehead) Figure 27.

## 6. Conclusions

This narrative review highlights the clinical utility of children’s drawings as an adjunctive diagnostic and therapeutic tool in pediatric headache management. Given young patients’ frequent difficulties in verbal symptom description, drawing provides an accessible medium for expressing pain characteristics, including location, intensity, and associated symptoms (e.g., nausea, photophobia, and aura manifestations). Current evidence demonstrates significant correlations between graphic representations and clinical diagnoses, with certain elements achieving >80% diagnostic accuracy. Additionally, serial drawings offer valuable longitudinal tracking of treatment response. Integrating this approach into clinical practice may enhance diagnostic precision while promoting a developmentally appropriate, child-centered, empathetic model of care.

## Figures and Tables

**Table 1:** Studies on drawings and headache diagnosis.

	Cohort	Age and Gender	Materials Methods	Results
Unruh et al. (1983) [14]	109	5–18 years; 66 females, 43 males	Children were asked to draw their pain and how they experience it. Drawings were analyzed for symbolic and emotional elements.	32% of children depicted actions or instruments causing pain, 19% personified the pain, 25% used abstract representations, and 11% localized the pain on the body. Children with migraine frequently depicted themselves relieving their pain (48%) compared to those with musculo-skeletal pain (31%). Red and black were the most dominant colors.
Stafstrom et al. (2002) [11]	226	4–19 years; 105 males and 121 females	Children created drawings depicting pain localization, intensity, and related emotions. Two pediatric neurologists analyzed the drawings against clinical diagnosis.	Headache drawings showed a sensitivity of 93% and a specificity of 83% compared to clinical diagnosis. A total of 90% included precise pain localization; 78% showed emotional content; 68% included metaphorical elements like lightning bolts linked to migraines.
Wojaczynska-Stanek et al. (2008) [16]	124	5–18 years; 68 females, 56 males	124 children (40 migraines, 47 tension headaches, 37 others) provided pain drawings, analyzed for patterns in localization, color, and symbolic elements.	Pain localization in 68% of drawings, symbolic elements in 25%, and 73% dark colors; migraines often had unilateral depictions.
Mosquera et al. (2008) [17]	48	5–19 years; gender not specified	Children drew their headache perceptions. Drawings were evaluated by a pediatric neurologist and compared with clinical findings.	75% depicted unilateral pain, 60% used symbolic elements (lightning bolts, tears), 80% used black or gray, correlating with migraine characteristics.
Mazzotta et al. (2015) [18]	67	6–14 years; gender not specified	67 children with headaches and 90 controls created drawings. Two child neuropsychiatrists blinded to clinical data analyzed the patterns.	78% of drawings localized pain; 48% included symbolic imagery (e.g., lightning bolts). Sensitivity for migraines: 85.71%; tension headaches: 81.48%.
Yilmaz et al. (2019) [19]	5	14–18 years; 3 females, 2 males	Adolescents with migraines and visual aura were asked to depict their symptoms, focusing on visual disturbances like zigzag lines and scotomas.	100% depicted visual aura symptoms, including zigzag lines and scotomas, confirming their diagnostic value for adolescents.
Garcia-Ron et al. (2024) [20]	132	12 years (mean); 61.1% females	Children with headaches drew their pain experiences without instructions. Neuropediatricians and neurologists assessed the drawings for diagnostic insights.	78.5% concordance for migraines and 78.6% for tension headaches; migraine features like aura and nausea showed 100% diagnostic match.

**Table 2:** Correlation between the clinical phenotypes and the graphic representation of pain.

Localization of pain	Unilateral: (Migraine–PPV 63.6%) [11] - Hand holding one side of the head - Scripts explaining the location of pain - Circles/arrows marking the precise location of pain - Objects/lines located on one side of the head - Sparkles on one side of the head Bilateral: (Migraine–PPV 55.6%) [11] - Hands holding both side of the head - Scripts explaining the location of pain - Circles/arrows marking the precise location of pain - Objects located on both side of the head - Circles/bandages all around the head
Quality of pain	Pulsating: (Migraine–PPV 83.2%) [11] - Heart - Hammer - Drums - Pendulum - Scripts or onomatopoeia (“pum pum”, “bam bam”) - Sharp objects hurting a spot on the head Pressing: - Stones/weights loading on the head - Clamps - Hands holding head - Gears Tightening: - Bandages/knots constricting the head - Head-binding material Stabbing: - Knives - Drills Burning: - Flames/candle/fire
Intensity of pain	(Migraine–PPV n.a.) Lower: - Smaller drawings - Location of pain outside the head - Brighter images - Blank expressions/smile Higher: - Larger drawings - Location of pain inside the head - Dark images - Color red/black - Sadness/pain expressions - Tears - Supine position - Scripts or onomatopoeia explaining the intensity of pain
Aggravation by or causing avoidance of routine physical activity	- Supine position/laying in bed - Scripts explaining the will of avoiding activity
Nausea	(Migraine–PPV 90.9%) [11] - Hands holding abdomen or stomach - Hands on mouth
Vomiting	(Migraine–PPV 90.9%) [11] - W.C. - Vomit - Hands holding abdomen or stomach
Photophobia	(Migraine–PPV 91.3%) [11] - Light sources (tv, sun, lights, windows, ...) crossed out - Scripts explaining the distress caused by lights
Phonophobia	(Migraine–PPV 80%) [11] - Noise sources (voices, drums, musical notes, horns, ...) crossed out - Scripts explaining the distress caused by noises/sounds
Visual aura	(Migraine–PPV 95%) [11] - Stars, colored spots (phosphenes) - Dark/black spots (scotomas) - Zigzag lines (fortification spectra) - Half objects/faces (hemianopsia) - Fog
Sensory aura	(Migraine–PPV n.a.) - Dots or crosses along the affected limb as a feeling of numbness
Aphasia or Disartria	(Migraine–PPV n.a.) - Scripts explaining the inability to produce sounds correctly and articulation impairment
Motor aura	(Migraine–PPV 63.6%) [11] - Darker half of the body - Scripts nearby arms/legs as “dead” or “dying” [36]
Brainstem aura	(Migraine–PPV n.a.) - Double vision/objects - Fog - Lines/circles/spirals around the head

Note: Migraine PPV = predictive positive value for that feature in Migraine; []: n. reference; n.a.: not available.

## Data Availability

No new data were created, and clinical data are unavailable due to privacy or ethical restrictions.

## Supplementary Materials

The following supporting information can be downloaded at: https://www.mdpi.com/article/10.3390/life15070996/s1, Figure S1: Glossary: Figurative symbol based on our children’s drawings and clinical meaning; Table S1: Characteristics of visual symptoms: differential diagnosis between migraine and epilepsy.

## Abbreviations

ICHD-3	International Classification of Headache Disorders
PPV	positive predictive value
IHS	International Headache Society
CSD	cortical spreading depression
