# Supplementary material for: Children’s Headache Through Drawings: A Narrative Review and a Portrait Gallery
Source: Life (Basel). 2025 Jun 23;15(7):996. doi: 10.3390/life15070996 (PMC12298764; doi:10.3390/life15070996)
Supplement: Supplementary file 1 [file life-15-00996-s001.zip › Glossary - Figurative symbol based on our children’s drawings and clinical meaning.pdf]

Glossary : Figurative symbol based on our children's drawings and clinical meaning

Clinical Meaning	Figurative Symbol
Quality pain	
Hammering Pain	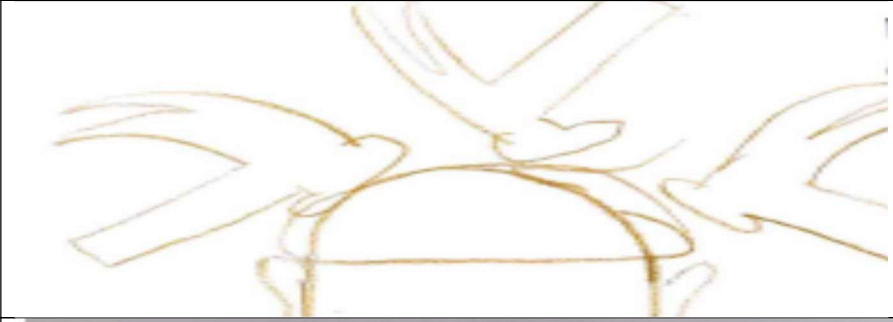
Pulsanting Pain	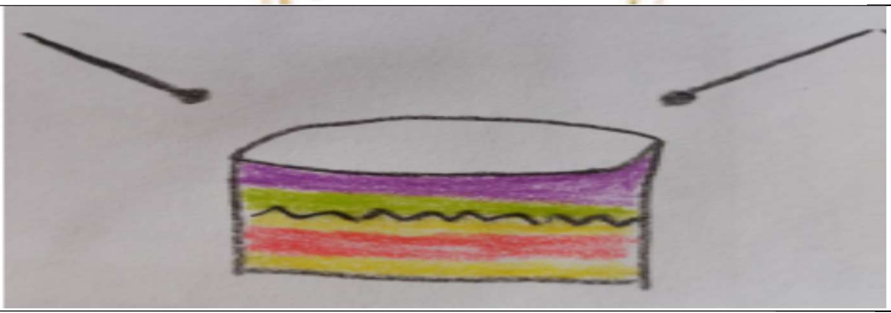
Pain with lightning bolts	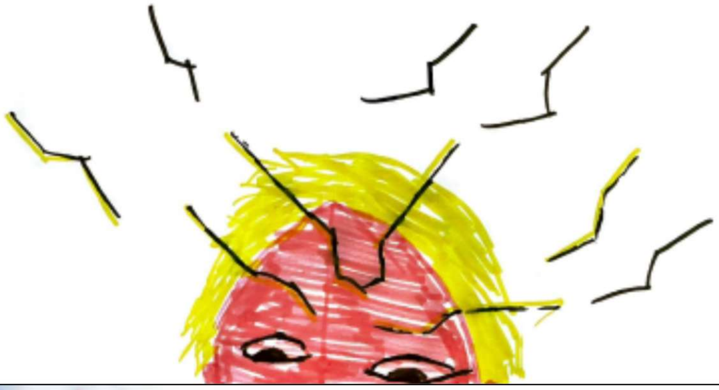
Burning , Constricting , Freezing Stabbing and Explosive Pain	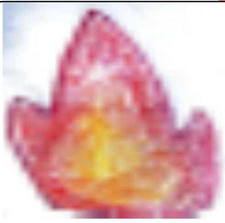 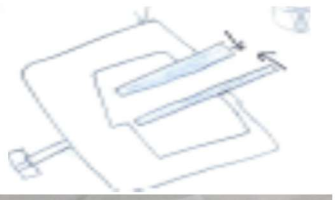 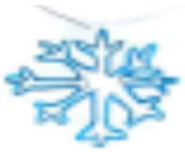 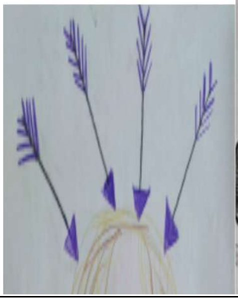 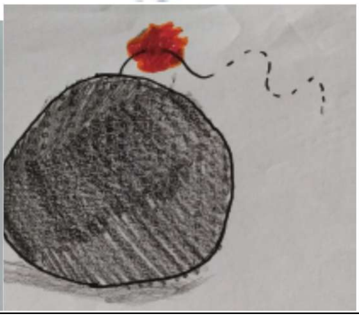

<table>
<tr><td>Intensity pain</td><td></td></tr>
<tr><td>Grading pain
Intensity</td><td>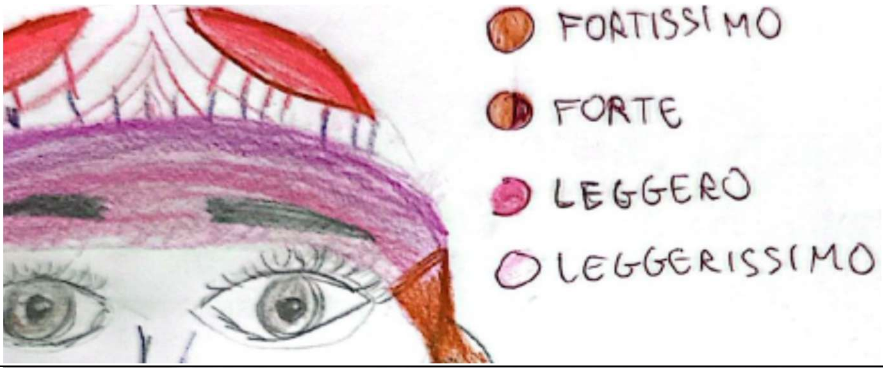
</td></tr>
<tr><td>Severe pain</td><td>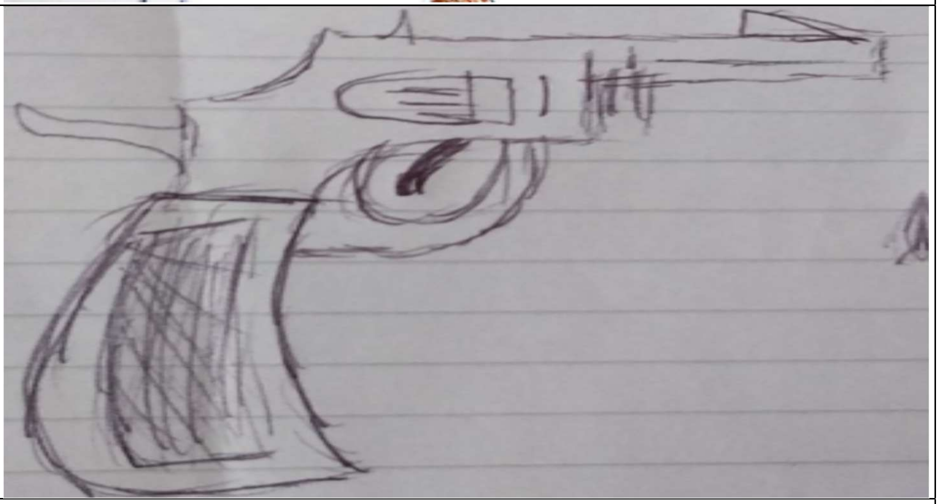</td></tr>
<tr><td>Localization</td><td></td></tr>
<tr><td>Unilateral pain</td><td>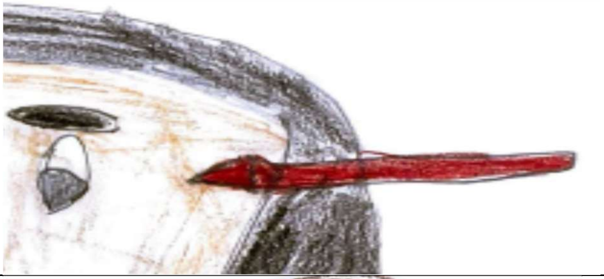</td></tr>
<tr><td>Bilateral Pain</td><td>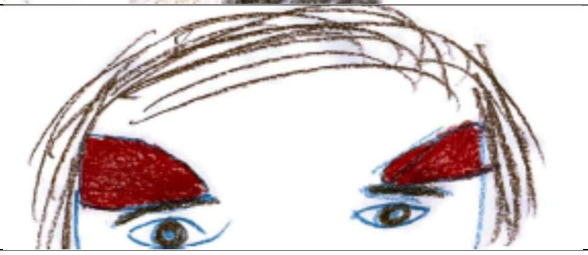</td></tr>
</table>

Frontal Pain	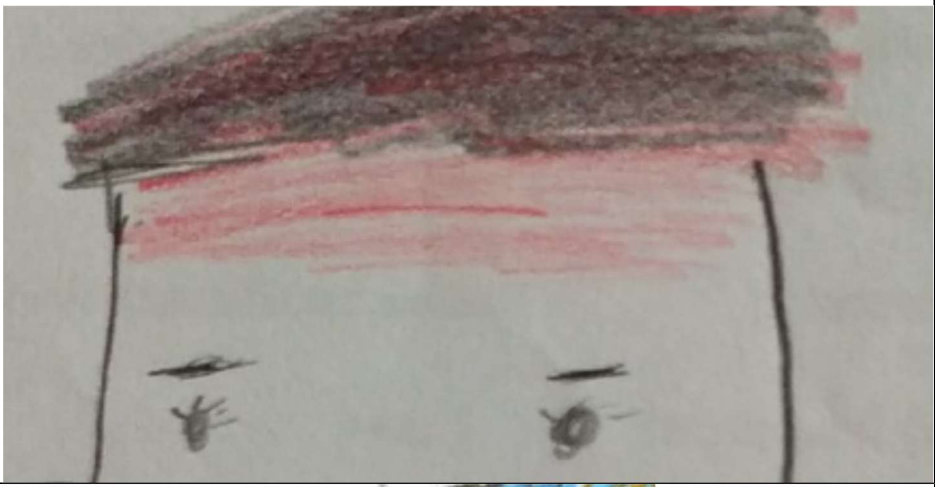
Trochlear Pain<br>Orbital Pain<br>Sovraorbital Pain	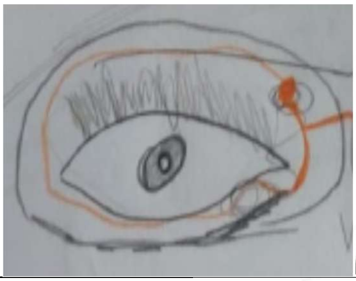 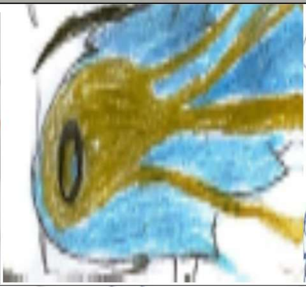 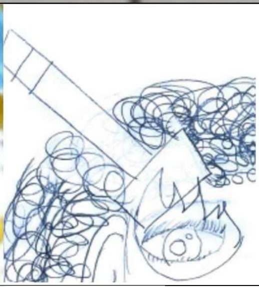
Facial Pain	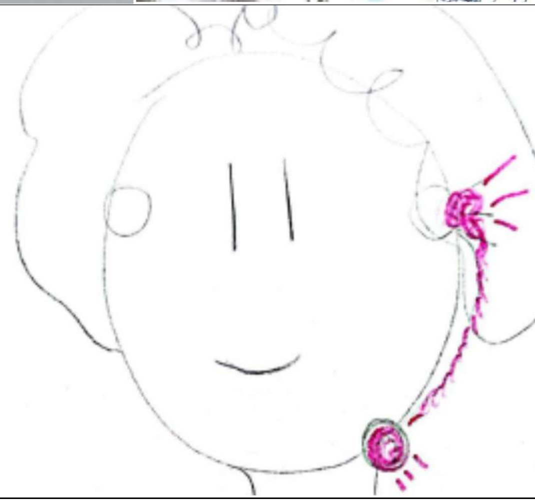
Nummular Headache	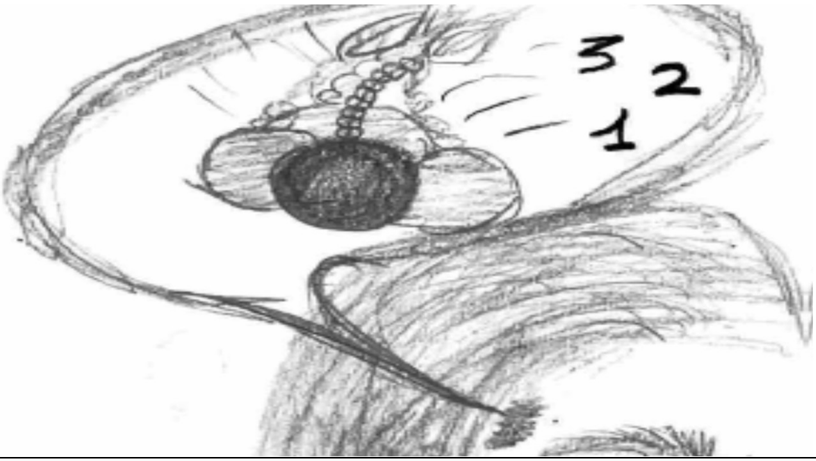

Associated Symptoms	
Vomit	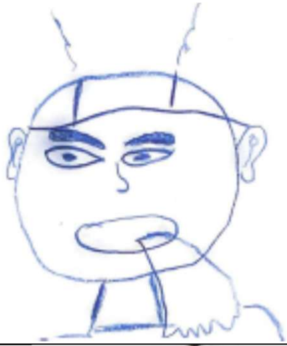 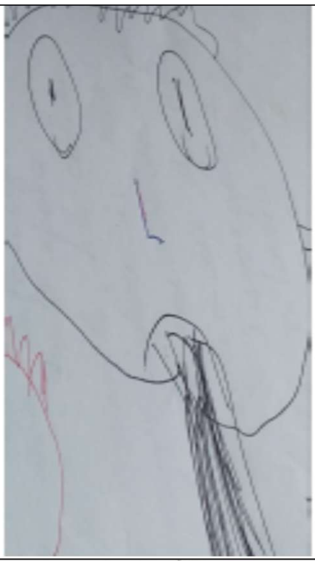
Nausea	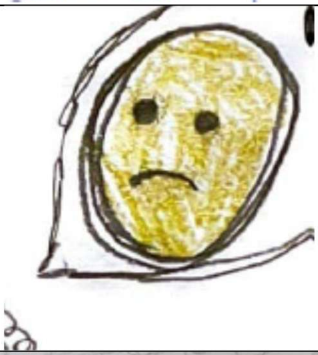 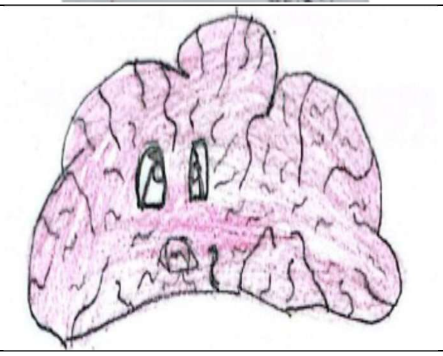
Photophobia	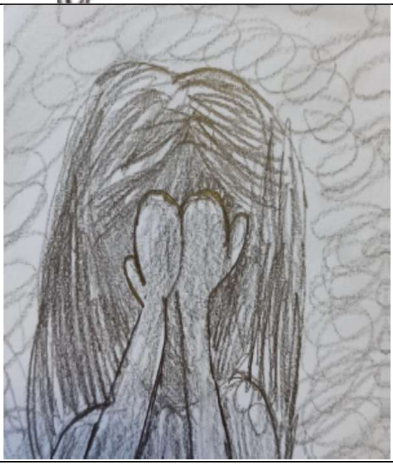 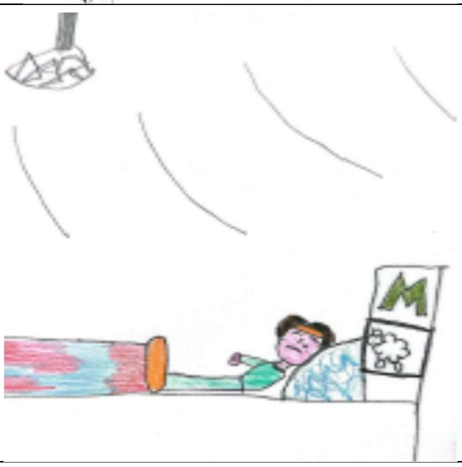

Phonophobia	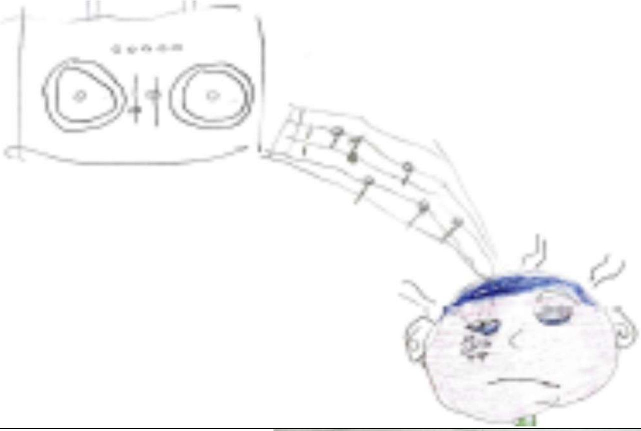
Brain Fog/ Confusion	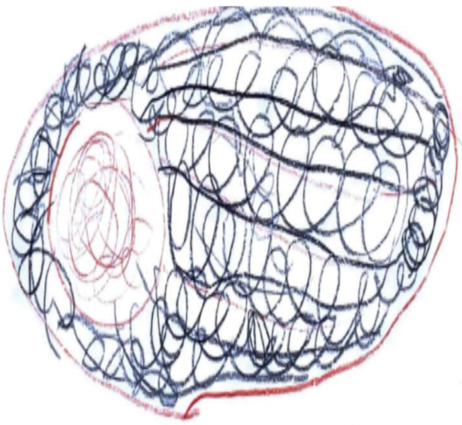 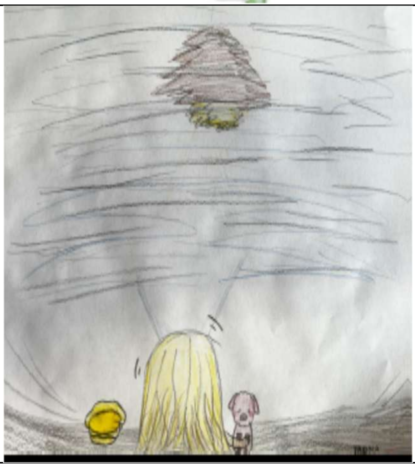
Aura	
Phosphenes	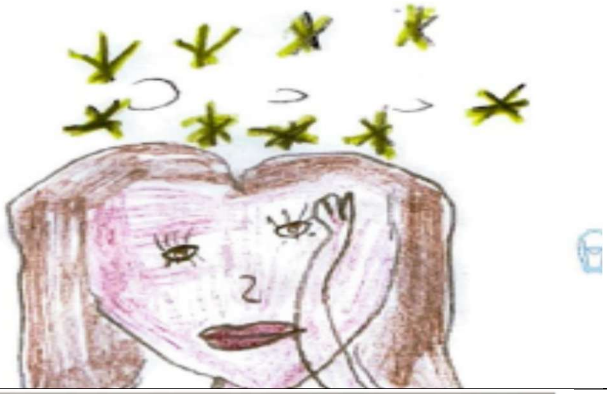
Colored balls	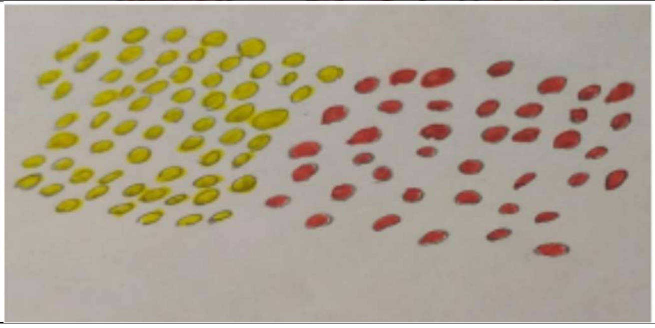

Stars and Scotomi	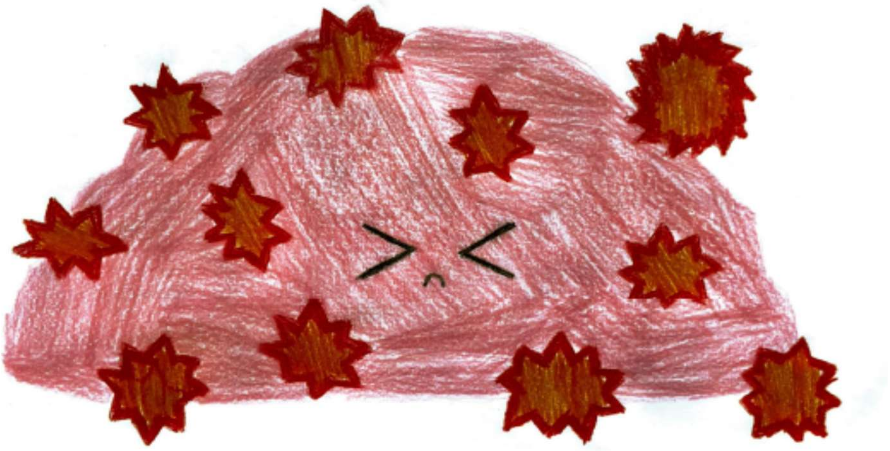
Spirals	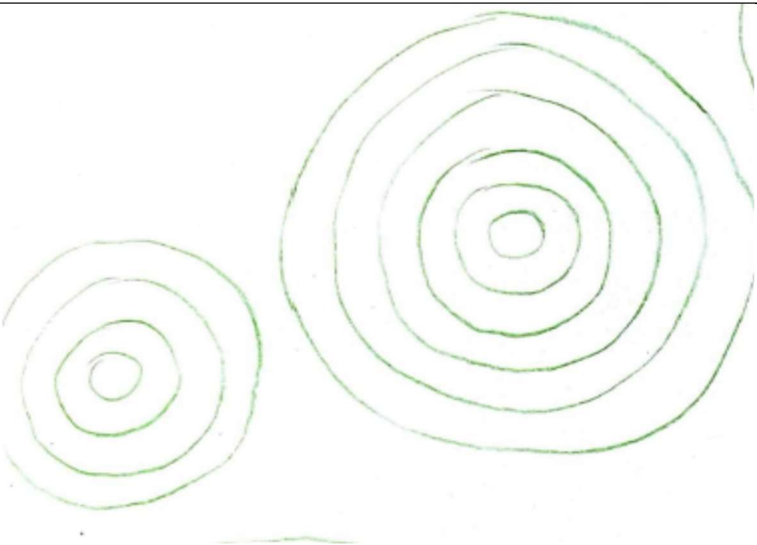
Zig Zag	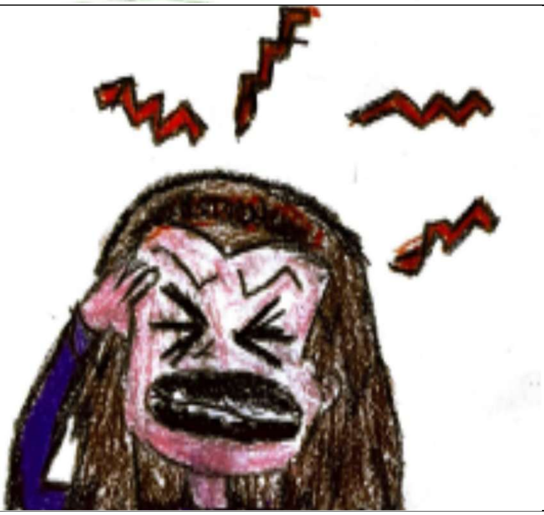

Diplopia	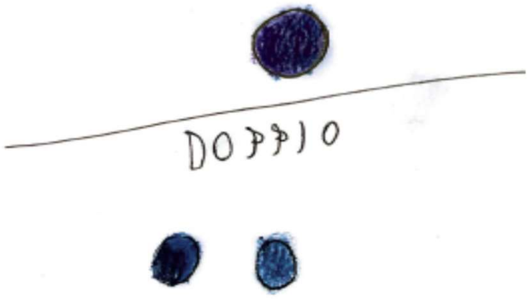
Hemianopsia	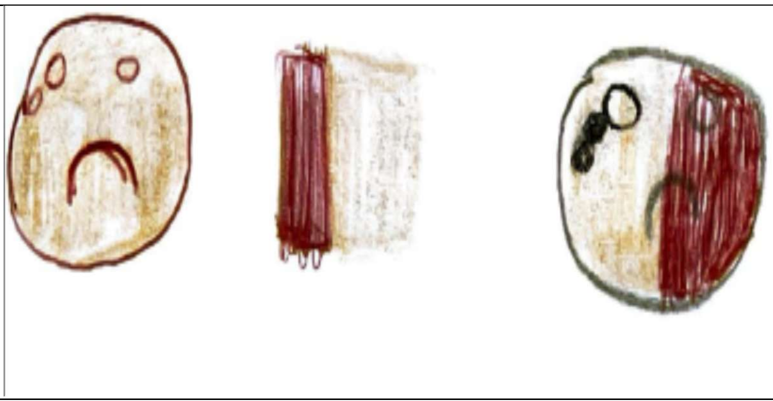
Disability/Relief	
Only Disability	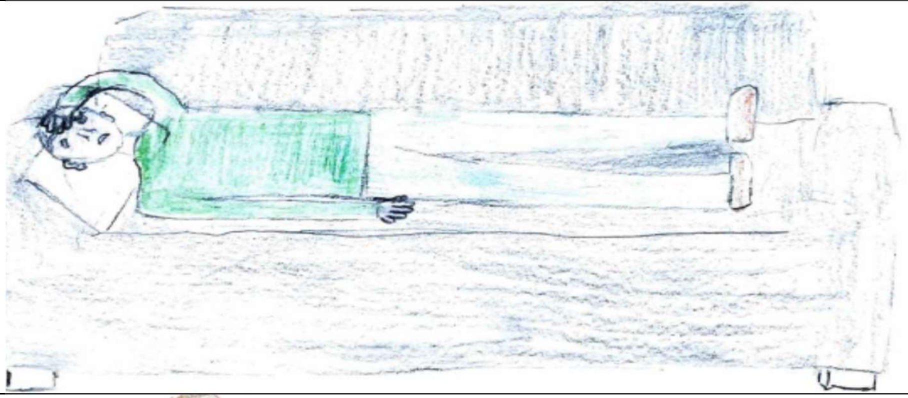
During/After	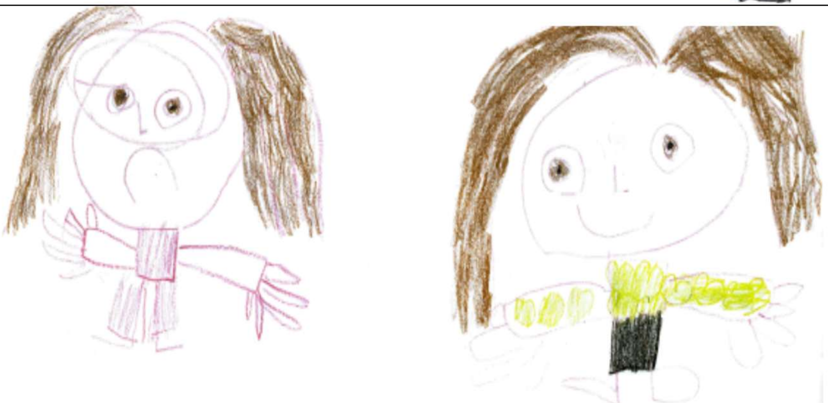

Before/During	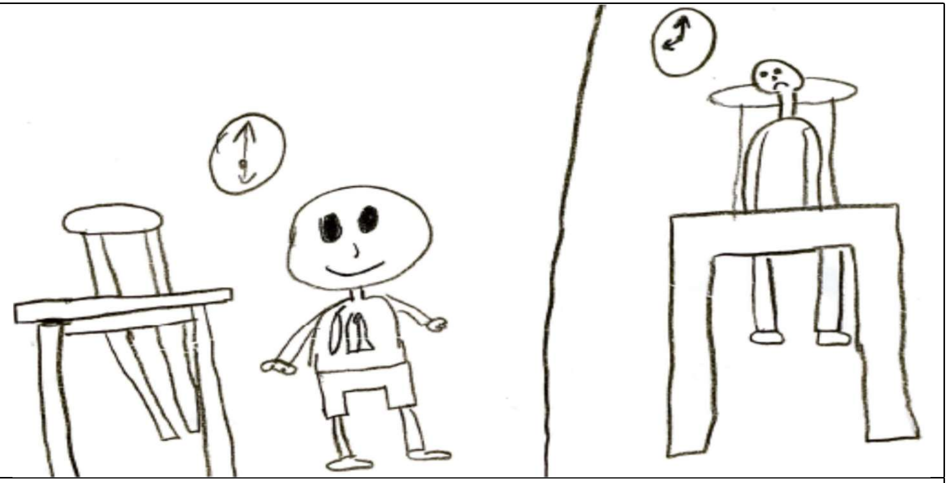
What Kids Say About Their Headaches	
?<br>“Why me?”<br>"Sometimes I get angry, but then I know I'll get over it!"	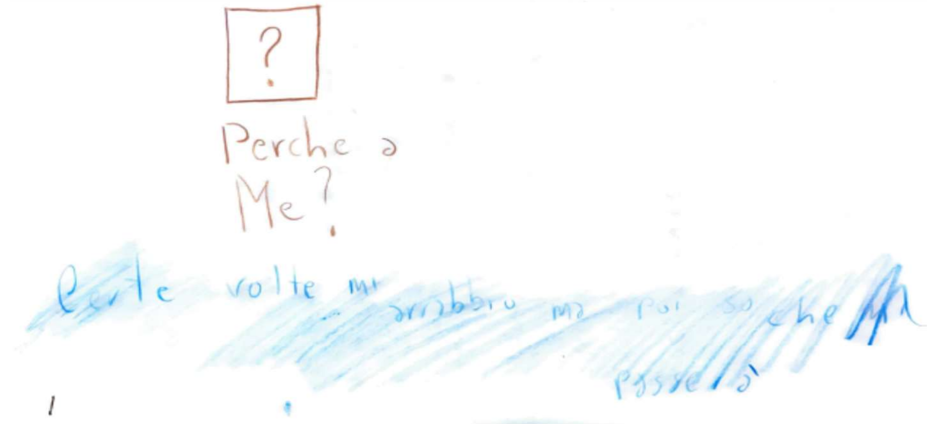
