# Supplementary material for: Children’s Headache Through Drawings: A Narrative Review and a Portrait Gallery
Source: Life (Basel). 2025 Jun 23;15(7):996. doi: 10.3390/life15070996 (PMC12298764; doi:10.3390/life15070996)
Supplement: Supplementary file 1 [file life-15-00996-s001.zip › Table supplementary.pdf]

Table S1. Characteristics of visual symptoms: differential diagnosis between migraine and epilepsy.

Symptoms			Migraine	Epilepsy	Ref (Auth; Journal, Year)
Negative		Scotoma<br>Monocular<br>Binocular	Frequent<br>Rare		Schwartz et al. ,**Eye Brain** 2012
		Blurred vision	Frequent	Rare	Petrusic et al. **JHP** 2014
		"Tunnel" vision	Frequently reported in children	Anecdotal in adults	Petrusic et al. **JHP** 2014
		Blindness<br>Monocular<br>Binocular	Consequence of scotoma; very rare occurrence in adults; female-predominance	2/3 of patients	Aldrich et al. **Epilepsia** 1989<br>Queiroz et al. **Cephalalgia** 2011<br>Rozen et al. **Headache** 2011
Positive	Simple	Bright light/unformed flashes of light/ star-shaped figures	Commonly reported; a centripetal/- fugal drift of the phosphenes	Horizontal drift stereotypic lateralization	Hartl et al. **Headach**e 2017
		Scintillating scotoma	Frequent	Rare	Petrusic et al. **JHP** 2014<br>Panyiotopoulos et al. **J.Neurl Neurosurg Psychiatr** 1999
		Zig-zag or jagged lines	Frequent		Petrusic et al. **JHP** 2014<br>Russel et al**. Brain** 1996<br>Queiroz et al. **Cephalalgia** 2011
		Black dots	A centripetal/- fugal drift of the phosphenes; relatively common	Horizontal drift stereotypic lateralization	Hartl et al. **Headache** 2017<br>Queiroz et al. **Cephalalgia 2011**
		Color dysgnosia<br>Color brightness	Frequent	No data	Petrusic et al. **JHP** 2014<br>Hadjikhani et al. **Vision** 2021
		Phosphenes (small bright dots)	A centripetal/- fugal drift of the phosphenes	Horizontal drift stereotypic lateralization	Hartl et al. Headache 2017
		Curved or circular lines	Relatively common		Queiroz et al. **Cephalalgia** 2011
		"Bean-like" forms like a crescent or C-shaped	Frequent		Queiroz et al . **Cephalalgi**a 2011
		Flickering light	A centripetal/- fugal drift of the phosphenes; Frequent		Russell et al. Brain 1996
		Palinopsia	No report in children	Frequent	Adcock et al. **Clin** Neurophysiol. 2021
		Polyopia	One report	rare	Raieli et al. **JHP** 2000
	Complex	Visual snow	Seven anecdotal cases; isolated, short-lasting	Two reports;	Simpson et al. **Ped Neurol** 2013

			visual snow syndrome	Polster et al. **Pediatrics** 2019
	Mosaic vision	Relatively common		Podoll et al. **Cephalalgia** 2001 Queiroz et al. **Cephalalgia** 2011]
	Fractured Vision	Relatively common		Podoll et al. **Cephalalgia** 2001
	Corona effect	Single or two extra contours, surrounding parts or the complete contours of an object, in black/white or in color, around perceived or illusory images	Not described	Podoll et al. **Cephalalgia** 2001
	Complex hallucinations	Rare	Frequent	
	Alice in Wonderland syndrome (micropsia, macropsia, teleopsia, metamorphopsia, pelopsia)	When AWS is associated with migraine, micropsia and teleopsia have most frequently been reported	Rare	Shevell et al. **Ped Neurol** 2012
	People, animals, landscape	Extremely rare	Rare	Akiyama et al. **BMJ case** 2019 Smith et al. **Arch Dis Child** 2013

Legend (Scientific journals in bold)- JHP: Journal of Headche and Pain; BMJ case: British Medical Journal case
